# A novel epigenetic CREB-miR-373 axis mediates ZIP4-induced pancreatic cancer growth

**DOI:** 10.1002/emmm.201302507

**Published:** 2013-07-16

**Authors:** Yuqing Zhang, Jingxuan Yang, Xiaobo Cui, Yong Chen, Vivian F Zhu, John P Hagan, Huamin Wang, Xianjun Yu, Sally E Hodges, Jing Fang, Paul J Chiao, Craig D Logsdon, William E Fisher, F Charles Brunicardi, Changyi Chen, Qizhi Yao, Martin E Fernandez-Zapico, Min Li

**Affiliations:** 1 Michael E. DeBakey Department of Surgery, Baylor College of MedicineHouston, TX, USA; 2 The Vivian L. Smith Department of Neurosurgery, the University of Texas Medical School at HoustonHouston, TX, USA; 3 Division of Biostatistics, School of Public Health, the University of Texas Health Science Center at HoustonHouston, TX, USA; 4 Department of Pathology, UT MD Anderson Cancer CenterHouston, TX, USA; 5 Department of Pancreatic and Hepatobiliary Surgery, Fudan University Shanghai Cancer CenterShanghai, China; 6 Department of Oncology, Shanghai Medical College, Fudan UniversityShanghai, China; 7 Pancreatic Cancer Institute, Fudan UniversityShanghai, China; 8 The Key Lab of Nutrition and Metabolism, Institute for Nutritional Sciences, Shanghai Institutes for Biological Sciences, Chinese Academy of SciencesShanghai, China; 9 Department of Molecular and Cellular Oncology, UT MD Anderson Cancer CenterHouston, TX, USA; 10 Department of Cancer Biology, UT MD Anderson Cancer CenterHouston, TX, USA; 11 Department of Surgery, David Geffen School of Medicine at UCLALos Angeles, CA, USA; 12 Schulze Center for Novel Therapeutics, Mayo ClinicRochester, MN, USA

**Keywords:** microRNA-373, pancreatic cancer, zinc, ZIP4

## Abstract

Changes in the intracellular levels of the essential micronutrient zinc have been implicated in multiple diseases including pancreatic cancer; however, the molecular mechanism is poorly understood. Here, we report a novel mechanism where increased zinc mediated by the zinc importer ZIP4 transcriptionally induces miR-373 in pancreatic cancer to promote tumour growth. Reporter, expression and chromatin immunoprecipitation assays demonstrate that ZIP4 activates the zinc-dependent transcription factor CREB and requires this transcription factor to increase miR-373 expression through the regulation of its promoter. miR-373 induction is necessary for efficient ZIP4-dependent enhancement of cell proliferation, invasion, and tumour growth. Further analysis of miR-373 *in vivo* oncogenic function reveals that it is mediated through its negative regulation of TP53INP1, LATS2 and CD44. These results define a novel ZIP4-CREB-miR-373 signalling axis promoting pancreatic cancer growth, providing mechanistic insights explaining in part how a zinc transporter functions in cancer cells and may have broader implications as inappropriate regulation of intracellular zinc levels plays an important role in many other diseases.

## INTRODUCTION

Zinc is an essential trace element functioning as a catalytic cofactor for multiple enzymes; and it is required to maintain the expression and structure of numerous signalling molecules and transcriptional regulators, many of which are involved in cancer growth and metastasis (Dufner-Beattie et al, 2003; Juhasz et al, 2003; Wang et al, 2002). Intracellular levels of zinc are maintained by two solute-linked zinc carrier families with antagonistic functions. ZnT transporters reduce cellular zinc through efflux, while the ZIP family increases intracellular zinc by promoting extracellular uptake. Aberrant expression of zinc transporters leading to altered intracellular zinc levels are involved in the pathogenesis of multiple cancers. For example, chemically induced mammary tumours are characterised by reduced expression of the ZnT1 and a 12-fold increase in intracellular zinc levels (Liuzzi & Cousins, 2004). Overexpression of ZIP6 increases zinc levels and associates with oestrogen-positive breast cancer and lymph node metastasis (Taylor et al, 2003), while ZIP10 is required for the invasive properties of breast cancer cells (Kagara et al, 2007). We have recently demonstrated a novel biological role for the zinc importer ZIP4 in pancreatic cancer (Li et al, [Bibr b16],[Bibr b17]). ZIP4 is overexpressed in human pancreatic cancer and promotes tumour growth and metastasis (Li et al, 2007). Although the biological effects of intracellular zinc and zinc transporters are clearly established in multiple malignancies, the molecular mechanism controlling this phenomenon remains elusive.

Here, using pancreatic cancer as a disease model, we have defined a novel oncogenic pathway downstream of zinc importer ZIP4 involving CREB-dependent induction of miR-373. This newly identified axis can promote pancreatic cancer growth both *in vitro* and *in vivo*. Further analysis demonstrated that the mechanism mediating this oncogenic effect was the miR-373 silencing of molecules with tumour suppressor activity including TP53 inducible nuclear protein 1 (TP53INP1), large tumour suppressor homolog 2 (LATS2) and CD44. We show that downregulation of these molecules increases pancreatic cancer growth. These results are of clinical importance since pancreatic cancer is a devastating disease ranking fourth in cancer-related deaths in the United States (Landis et al, 1998). Most patients at diagnosis have aggressive, metastatic disease with a median life expectancy of less than 1 year. This malignancy is incurable largely due to lack of therapeutic approaches and insufficient knowledge of its biology. Therefore, it is imperative to define the mechanism mediating initiation, maintenance and progression of this deadly disease. Together, these findings define a novel pathway activated by the dysregulated intracellular zinc level leading to increased pancreatic tumour growth that may help the design of future clinical studies for this dismal disease as well as other diseases with aberrant intracellular levels of zinc.

## RESULTS

### ZIP4 induces the expression of miR-373 in pancreatic cancer

**Figure 1:**
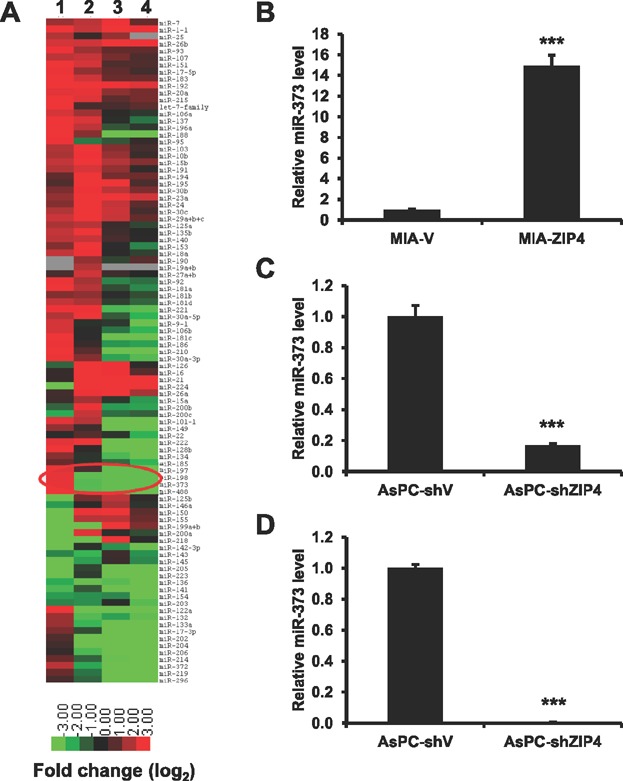
ZIP4 induces the expression of miR-373 in pancreatic cancer **A.** Numerous microRNAs are differentially expressed in ZIP4 overexpressing and silencing cells and xenografts. A heat-map is shown in which colours represent relative levels of microRNAs with the brightest red indicating those with the highest ratio and green depicting the lowest ratio for the indicated comparisons. Lane 1: MIA-ZIP4 versus MIA-V cells; lane 2: AsPC-shZIP4 versus AsPC-shV cells; lane 3 and 4: AsPC-shZIP4 versus AsPC-shV xenografts #1 and #2.**B–D.** miR-373 expression in (B) MIA-ZIP4 cells, (C) AsPC-shZIP4 cells, and (D) AsPC-shZIP4 orthotopic xenografts (*P* < 0.0001, *t*-test, *n* = 3). Data were expressed as the mean ± SD of triplicate values.

To define the molecular mechanisms of ZIP4-mediated pancreatic cancer growth, we performed an expression profile analysis in our previously described ZIP4-overexpressed (MIA-ZIP4) or silenced (AsPC-shZIP4) pancreatic cancer cells and xenografts (Li et al, [Bibr b16],[Bibr b17]). Interestingly, miR-373 was prominently upregulated by ZIP4 overexpression in each of the pairwise comparisons (Fig 1A). Conversely, knockdown of ZIP4 decrease the expression of this miRNA (Fig 1A). Further validation revealed a statistically significant effect on miR-373 expression *in vitro* (Fig 1B and C) and *in vivo* (Fig 1D) with a fold change ranging from 6 to over 200-fold. Similar results were observed in MIA-siZIP4 and AsPC-ZIP4 cells (Supporting Information Fig S1A and B). To determine the clinical relevance of these findings, we also examined the expression of ZIP4 and miR-373 in human pancreatic cancer specimens and found a positive correlation between ZIP4 and miR-373 expression (Supporting Information Fig S1C–E).

To confirm the role of zinc ion as a mediator of ZIP4-induced miR-373 upregulation, we depleted the intracellular zinc in both MIA PaCa-2 and AsPC-1 cells with the zinc chelator *N*,*N*,*N*′,*N*-tetrakis (2-pyridylmethyl) ethylenediamine (TPEN), and added low concentration of zinc in the culture medium (1 µM ZnCl_2_ for MIA PaCa-2 cells and 0.5 µM ZnCl_2_ for AsPC-1 cells, respectively). Removal of zinc by TPEN treatment significantly decreased the miR-373 level in cells overexpressing ZIP4 and has minimal impact on miR-373 in control MIA-V cells which have low ZIP4 expression (Supporting Information Fig S1F). This was further supported by RNAi experiments, ZIP4 silencing causes decreased zinc induction of miR-373 (Supporting Information Fig S1G). Thus, together these results and our previous study on zinc-dependent cell proliferation (Li et al, 2007) support a role for zinc as an intermediate signalling molecule regulating ZIP4 effects in pancreatic cancer.

**Figure 2:**
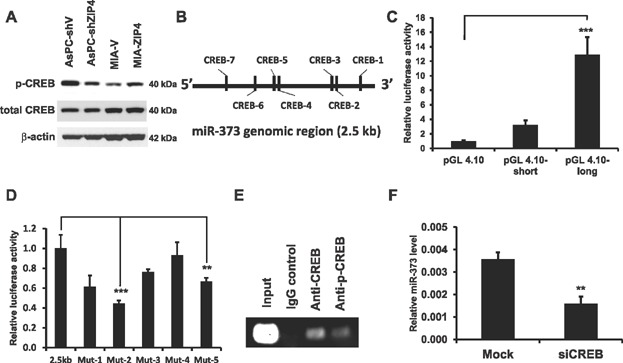
ZIP4 induces the expression of miR-373 through activation of the transcription factor CREB CREB phosphorylation was downregulated in AsPC-shZIP4 and upregulated in MIA-ZIP4 cells.Schematic structure of the 2.5 kb genomic region upstream of miR-373. There are seven potential CREB binding sites in this region.Promoter activity. The promoter reporter construct was co-transfected with pRL-TK into AsPC-1 cells, the promoter activity was determined by a chemiluminescence reader (significant *p*-value <0.0001, *t*-test, *n* = 4).Mutational analysis of miR-373 promoter. The wild type and mutant promoter constructs were co-transfected with pRL-TK into AsPC-1 cells. The promoter activity was determined by a luciferase as described above (from left to right, significant *p*-value *=* 0.0002, 0.0035; *t*-test, *n* = 4).ChIP binding assay with anti-CREB and anti-p-CREB antibody in AsPC-1 cells confirmed the binding of CREB to the miR-373 promoter region.Silencing of CREB leads to decreased expression of miR-373 in pancreatic cancer cells. AsPC-1 cells were transfected with siRNA against CREB (Mission siRNA from Sigma). The miR-373 level was determined by real time PCR (*p* *=* 0.0014; *t*-test, *n* = 3). Data were expressed as the mean ± SD of triplicate values.

Further analysis identifies the transcription factor cAMP response element-binding protein (CREB) as a mediator of this phenomenon. ZIP4 regulates the activation of CREB in pancreatic cancer cells. MIA-ZIP4 showed increased phosphorylation of CREB compared to the control cells (Fig 2A). Conversely, activation of this transcription factor was significantly suppressed in AsPC-1 cells expressing a shRNA targeting ZIP4 (Fig 2A and Supporting Information Fig S2A). To determine if miR-373 is a transcriptional target of CREB, we examined the effect of ZIP4 on miR-373 promoter constructs containing wild-type and deletion mutations in predicted CREB binding sites. As shown in Fig 2B (labelled 5′ → 3′) and Supporting Information Fig S2B and C, there are multiple putative CREB binding sites upstream of miR-373. Deletion of predicted CREB binding sites #2 and #6 (Mut-2 and Mut-5) within the miR-373 promoter region decreased ZIP4-induced luciferase activity in AsPC-1 cells (Fig 2C and D) as well as MIA PaCa-2, PL45, and HEK293 cells (Supporting Information Fig S2D–G). As effect of mutations in the other candidate binding sites showed variable results throughout all cell lines, especially in pancreatic cancer cells, and in some cases it did not show statistical differences with the control group (Supporting Information Fig S2E, F and G). Therefore, we have decided to focus our studies on Mut-2 and Mut-5 as the CREB-responsive elements of this promoter. Chromatin immunoprecipitation assay (ChIP) confirmed the binding of CREB to the miR-373 promoter (Fig 2E and Supporting Information Fig S2H). Finally, we demonstrate that the siRNA silencing of CREB significantly reduces the expression of miR-373 in pancreatic cancer AsPC-1 cells with high endogenous ZIP4 (Fig 2F). Further analysis in both MIA-V and MIA-ZIP4 cells shows a significant reduction of ZIP4-dependent miR-373 promoter activation in cells transfected with a siRNA targeting CREB, suggesting that not just basal miR-373 expression requires CREB, but ZIP4-dependent induction of miR-373 relies on an active CREB transcription factor (Supporting Information Fig S2I). Together these results define miR-373 as a target of ZIP4 signalling in pancreatic cancer and indicate the requirement of intact CREB binding sites to modulate miR-373 promoter activity.

### miR-373 is required by ZIP4 to promote pancreatic cancer growth and metastasis

**Figure 3:**
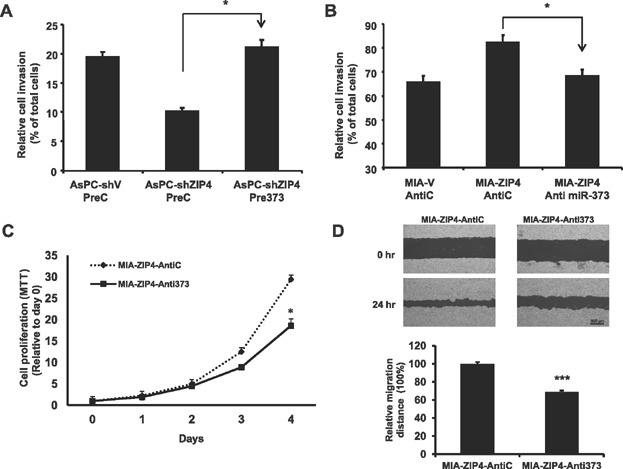
miR-373 promotes ZIP4-mediated pancreatic cancer cell invasion and proliferation Relative cell invasion in AsPC-shV and AsPC-shZIP4 cells transfected with precursor miR-373 (Pre miR-373) and precursor control miRNA (Pre Ctrl) (significant *p*-value *=* 0.012, *t*-test, *n* = 3).Cell invasion in MIA-V and MIA-ZIP4 cells transfected with antisense miR-373 (Anti miR-373) and control (Anti Ctrl) oligos (significant *p*-value *=* 0.034, *t*-test, *n* = 3).Cell proliferation in miR-373 blocking MIA-ZIP4 cells. Cell proliferation was assessed by MTT assay and compared with day 0 value (*p* *<* 0.0001, *t*-test, *n* = 3).Wound healing assay. A representative view of wound healing was captured and recorded at 0 and 24 h. The scale bar in the image is 500 µm (*p* *<* 0.0001, *t*-test, *n* = 8). All data are mean ± SE.

To investigate the biological role of miR-373 in ZIP4-mediated pancreatic cancer growth, we performed *in vitro* (cell invasion, proliferation, and migration assays) and *in vivo* studies. As shown in Fig 3A, transfection of miR-373 precursor significantly increased the invasiveness of AsPC-shZIP4 cells, indicating that the decreased cell invasion caused by ZIP4 silencing can be rescued by increasing miR-373 levels. Conversely, inhibition of miR-373 by antisense oligonucleotide reduced ZIP4-induced cell invasion (Fig 3B). These data strongly indicate that ZIP4 and miR-373 are not only correlated in expression levels but are functionally related. To further validate this functional interplay between ZIP4 and miR-373, we have used lentivirus-mediated antisense miR-373, and found that inhibition of miR-373 in MIA-ZIP4 cells reduced proliferation and migration compared with control-infected cells (Fig 3C and D). Furthermore, we have shown that blocking miR-373 caused cell cycle arrest at G1 phase without significant change in apoptotic rate in cells overexpressing ZIP4 (Supporting Information Fig S3A–C). Similar experiments were performed in MIA-V cells showing unchanged cell proliferation and migration upon miR-373 blocking, indicating that decreased miR-373 is not having a general growth and migration inhibition effect independent of ZIP4 (Supporting Information Fig S3D and E). Together these results indicate a role for miR-373 as a mediator of ZIP4-induced pancreatic cancer growth.

**Figure 4:**
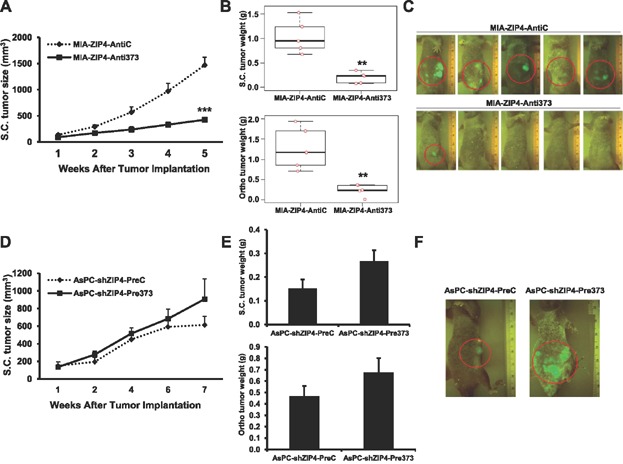
miR-373 mediates ZIP4-induced pancreatic cancer growth in a xenograft mouse model Subcutaneous tumour growth curve. MIA-ZIP4-AntiC and MIA-ZIP4-Anti373 cells (3 × 10^6^) were subcutaneously inoculated into the right flank of nude mice (*n* = 5). Tumour size was measured weekly. Tumour volume was calculated by the formula: tumour volume [mm^3^] = (length [mm]) × (width [mm])^2^ × 0.52 (*p* *=* 0.0002, *t*-test, *n* = 5).Boxplot charts of the s.c. and orthotopic tumour weight data overlaid with individual mice data (the individual data points are randomly jittered horizontally to avoid the completely overlapped data). The s.c. and orthotopic tumour weights were measured after the mice were euthanised at 5 weeks (Both charts have *p* = 0.008 using two-sided Wilcoxon rank sum test, *n* = 5).Group picture. Mice pictures were taken using Illumatool fluorescence imaging system at 5 weeks after tumour implantation.Subcutaneous tumour growth curve. AsPC-shZIP4-PreC and AsPC-shZIP4-Pre373 cells (3 × 10^6^) were subcutaneously inoculated into the right flank of nude mice (*n* = 5). Tumour size was measured weekly (*p* *=* 0.5, *t*-test, *n* = 5).S.C. and orthotopic tumour weight (*p* = 0.09, *t*-test, *n* = 5). All data are mean ± SE.One representative AsPC-shZIP4-PreC and AsPC-shZIP4-Pre373 mouse. Pictures were taken 5 weeks after tumour implantation.

To elucidate the effect of miR-373 on tumour growth *in vivo*, we generated GFP-positive stable cell lines to monitor tumour progression. In these cells we overexpressed or silenced miR-373 and used them in two complementary *in vivo* models: subcutaneous (s.c.) and orthotopic xenografts (Supporting Information Fig S4A–C). We found that inhibition of miR-373 significantly reduced tumour size (71% reduction) and tumour weight (81% reduction) in the s.c. model (Fig 4A and B), and reduced the primary tumour weight (81% reduction) in the orthotopic model (Fig 4B) with 20% mice tumour-free, compared with the control group, in which all mice had large tumours (Fig 4C). Conversely, overexpression of miR-373 in AsPC-shZIP4 cells increased tumour size (48% increased) and tumour weight (75% increase) in the s.c. model (Fig 4D and E), and increased the primary tumour weight (44% increase) in the orthotopic model compared with the control group, although the difference did not reach a statistical significance due to the high variability in the sample sets (Fig 4E and F). Furthermore, overexpression of miR-373 in AsPC-shZIP4 cells also caused increased incidence of ascites, jaundice, loss of body weight, and liver metastasis of the nude mice (Data not shown). These data indicate that miR-373 axis is a mediator of pancreatic cancer tumour growth and metastasis induced by this zinc importer.

### TP53INP1, LATS2, and CD44 are targets of miR-373 in pancreatic cancer

**Figure 5:**
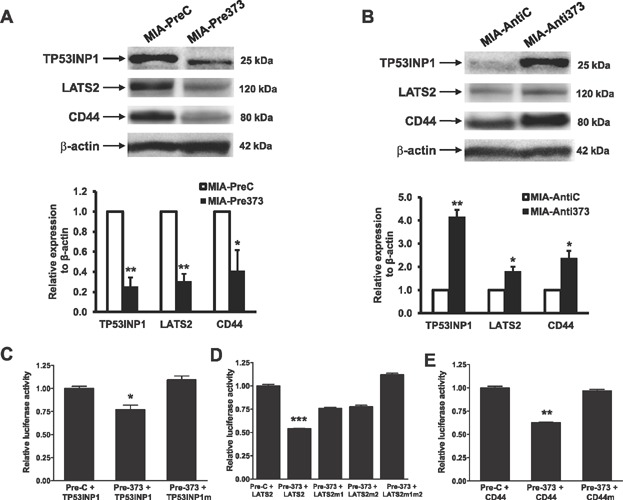
TP53INP1, LATS2 and CD44 are miR-373 targets in pancreatic cancer **A,B.** TP53INP1, LATS2 and CD44 expression levels were examined by Western blot in MIA PaCa-2 cells transiently transfected with Pre-miR-373 plasmid (A) (from left to right, *P* = 0.0049, 0.0037, 0.0104; *t*-test, *n* = 3) and Anti-miR-373 (B) (from left to right, *P* = 0.0031, 0.0214, 0.0173; *t*-test, *n* = 3).**C–E.** For the luciferase activity assay, wild type or mutant 3' UTR reporter vectors of (C) TP53INP1 (significant *P* value =0.0118, *t*-test, *n* = 3), (D) LATS2 (significant *P* value <0.0001, *t*-test, *n* = 3) and (E) CD44 (significant *P* value =0.0021, *t*-test, *n* = 3) were co-transfected into HEK293 cells together with the plasmid that expresses miR-373 precursor (Pre373) or vector control (PreC). The reporter gene activity was determined 48 h post-transfection.

Given the involvement of miR-373 in mediating ZIP4-dependent pancreatic tumour growth, we next investigated potential target genes regulated by miR-373. Using common prediction algorithms (TargetScan, MicroCosm Targets and PicTar) we have identified TP53INP1, LATS2, and CD44 as putative targets for miR-373. Initially, we analysed each of these genes in our cell lines with modulated ZIP4 expression and consequently miR-373 levels. Given that microRNAs are negative regulators of gene expression, *bona fide* targets of a specific microRNA would be predicted to be downregulated when the microRNA is highly expressed. As shown in Fig 5A and B, TP53INP1, LATS2 and CD44 levels were significantly reduced in MIA PaCa-2 cells transfected with Pre-miR373; while all three proteins were increased in MIA PaCa-2 cells transfected with Anti-373, indicating a direct post-transcriptional regulation of those three target genes by miR-373. Furthermore, all three proteins were reduced in MIA-ZIP4 cells, in which both ZIP4 and miR-373 levels are high, compared with MIA-V control cells. Conversely, their levels were significantly increased in AsPC-shZIP4 cells, in which both ZIP4 and miR-373 levels are low, compared to the control AsPC-shV cells (Supporting Information Fig S5A and B). Further experimentation indicate that silencing the miR-373 activator, CREB, in pancreatic cancer cells also leads to increased expression of TP53INP1 and CD44, respectively (Supporting Information Fig S5C and D). Luciferase reporter assays were performed for each of the three wild-type UTRs as well as mutant constructs where the putative miR-373 binding sites were abolished by site directed mutagenesis (Supporting Information Fig S5E–H). Unlike TP53INP1 and CD44 that have a single predicted miR-373 site in their 3′ UTR, the LATS2 3′ UTR contains two putative miR-373 sites that were mutated together or singly. For each wild-type 3′UTR target gene reporter, transfection of Pre-miR-373 significantly decreased luciferase activity and this repression required intact miR-373 binding sites (Fig 5C–E). These data indicate that TP53INP1, LATS2 and CD44 are direct targets of miR-373 in pancreatic cancer, and provide experimental evidence of the mechanism by which the ZIP4-miR-373 pathway promotes pancreatic tumour growth through the inhibition of key tumour suppressor genes.

**Figure 6:**
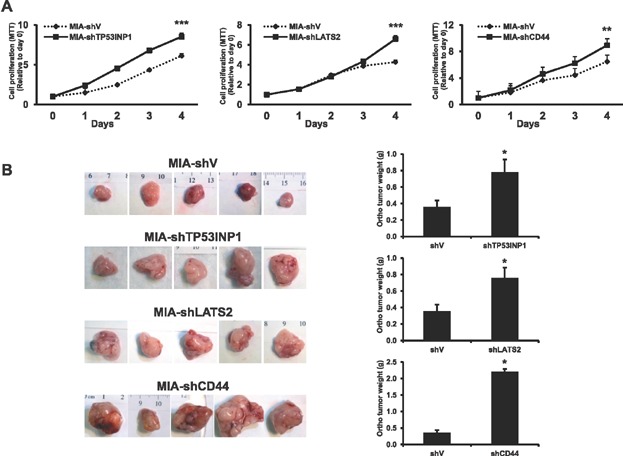
miR-373 target genes mediate ZIP4-induced pancreatic cancer growth Cell proliferation as determined by MTT assays in MIA-shTP53INP1, MIA-shLATS2, and MIA-shCD44 cells in comparison to day 0 value (from left to right, *p* *<* 0.0001, *<*0.0001, =0.0004; *t*-test, *n* = 5).Orthotopic tumour growth. Primary tumours were removed and five representative pictures from each group were shown. Tumour weight was measured after the mice were euthanised at 4 weeks (from top to bottom, *p* = 0.0299, 0.0136, 0.0158; *t*-test, *n* = 10–12). All data were expressed as the mean ± SEM.

**Figure 7:**
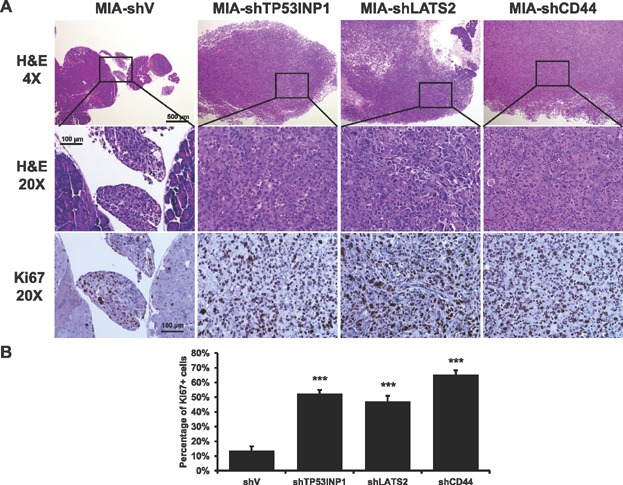
miR-373 target genes mediate ZIP4-induced pancreatic cancer metastasis H&E and Ki67 staining of the orthotopic xenografts from MIA-shTP53INP1, MIA-shLATS2 and MIA-shCD44 groups.Quantification of Ki67 staining of the orthotopic xenografts. The scale bars are 500 µm (4×) and 100 µm (20×) (*p* *<* 0.0001, *t*-test, *n* = 5).

To investigate the functional involvement of TP53INP1, LATS2 and CD44 in pancreatic cancer growth, shRNA-directed knockdown of TP53INP1, LATS2 and CD44 was performed and the effects on cell proliferation, migration and tumour growth were measured. MIA PaCa-2 and BxPC-3 cells were stably transfected with TP53INP1, LATS2, CD44 and negative control shRNAs, and synchronised by serum starvation for 24 h. In both MIA PaCa-2 and BxPC-3 cells, TP53INP1, LATS2 and CD44 knockdown cell lines showed increased cell proliferation compared with the control cells as determined by MTT (Fig 6A, Supporting Information Fig S6A and B). In addition, MIA-shLATS2 and BxPC-shLATS2 cells showed increased cell migration compared with the control cells (Supporting Information Fig S6C). Therefore, repression of TP53INP1, LATS2 and CD44 revealed that each gene plays an important role in controlling pancreatic cancer growth. We further investigated the function of the miR-373 target genes TP53INP1, LATS2 and CD44 *in vivo* by using an orthotopic xenograft model in nude mice. We found that silencing of TP53INP1, LATS2 and CD44 in MIA PaCa-2 cells significantly increased tumour growth compared with the vector control cells (Fig 6B), indicating a *bona fide* tumour suppressor function in pancreatic cancer cells. The primary orthotopic tumour weight was increased by 2.2, 2.1 and 6.2-fold in TP53INP1, LATS2 and CD44 silenced MIA PaCa-2 cells, respectively. Furthermore, mice with TP53INP1, LATS2 and CD44 knockdown cells showed peritoneal dissemination, and various degrees of liver, spleen and colon metastasis, and severe abdominal ascetic fluid (Supporting Information Fig S7), although the difference between mice with TP53INP1, LATS2 and CD44 knockdown cells and control group is not statistically significant except for colon metastasis. Tumours from orthotopically injected mice were removed and processed for further histological and immunohistochemical analysis. As shown in Fig 7, the majority of the tumours from the TP53INP1, LATS2 and CD44 silencing group were poorly differentiated, and the tumour area percentage were more than 80%, while most of the tumours from the MIA-V control group were well differentiated, and the tumour area percentage were less than 20%. Furthermore, tumours from the TP53INP1, LATS2 and CD44 silencing group showed increased cell proliferation as indicated by the strong staining of Ki67, compared with that of the MIA-V group. Those results indicate that repressing of TP53INP1, LATS2 and CD44 led to an increased tumour growth and its silencing may contribute to ZIP4/miR-373 induced pancreatic cancer growth.

## DISCUSSION

**Figure 8:**
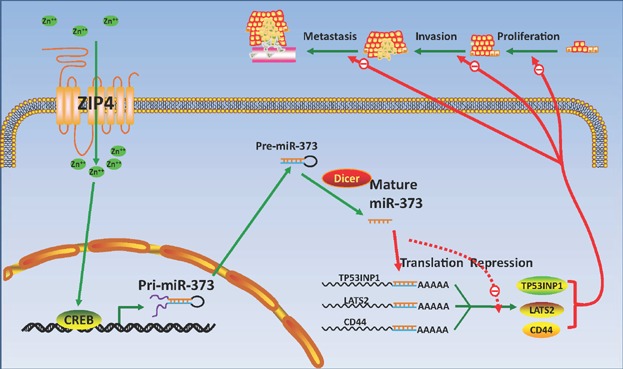
Working model for ZIP4/miR-373-mediated pancreatic cancer growth ZIP4 overexpression causes increased activity of miR-373 promoter through CREB, which leads to repressed expression of miR-373 target genes TP53INP1, LATS2 and CD44, resulting in increased cell proliferation, migration and tumour progression.

The current study identified a novel ZIP4-CREB-miR-373 signalling axis that promotes pancreatic cancer growth, through silencing on key tumour suppressor molecules including TP53INP1, LATS2 and CD44 (Fig 8). Our results suggest that ZIP4/miR-373 pathway can serve as a new therapeutic target for pancreatic cancer, a disease characterised by exceptionally poor prognosis. In addition, this study provides a comprehensive mechanism for ZIP4-mediated pancreatic cancer growth involving CREB-dependent transcription, enhancement of oncogenic miR-373 expression, and reduction in key miR-373 regulated targets, including the tumour suppressor genes TP53INP1, LATS2 and CD44. The information gained from this research may have important clinical implications for patients with pancreatic cancer as well as other cancer types associated with elevated intracellular zinc levels, and may also have clinical impact on other diseases in which zinc level is dysregulated.

Previous studies have shown that zinc plays an important role in cell proliferation possibly through influencing the DNA synthesis and cell cycle progression (Chesters & Boyne, 1991; King & Cousins, 2005; Lee et al, 2003; Tang et al, 2006). Our previous study also demonstrates that overexpressed ZIP4 increases pancreatic cancer cell proliferation under zinc deficient conditions (Li et al, 2007). However, high zinc level is toxic to the cells, and causes apoptosis (Kim et al, 2000). Therefore, cells must have a fine-balanced homeostasis to maintain the intracellular zinc level stable through the activity of zinc transporters. The role of zinc and zinc transporters in cancer such as breast cancer, prostate cancer, and hepatocellular carcinoma (HCC) has been clearly established. Zinc importers promote cell proliferation, migration, and metastasis in breast cancer and HCC, but have an inhibitory role in prostate cancer (Golovine et al, 2008; Kagara et al, 2007; Makhov et al, 2009; Taylor et al, 2003; Weaver et al, 2010).

Our group has recently demonstrated a novel biological role for the zinc importer ZIP4 in pancreatic cancer (Li et al, [Bibr b16],[Bibr b17]; Yang et al, 2013; Zhang et al, 2010b). We have shown that ZIP4 is a major zinc importer upregulated in pancreatic cancer, and promotes cancer growth. Overexpression of ZIP4 may cause activated IL-6/STAT3 pathway and also led to increased VEGF and MMP expression, however, the detailed mechanism of how ZIP4 overexpression causes the activation of the downstream signalling pathways to promote pancreatic cancer growth is not clear. In this study, we report a novel mechanism where increased zinc levels mediated by a zinc importer ZIP4 transcriptionally induce miR-373 in a CREB-dependent manner in pancreatic cancer cells. These results define a novel ZIP4-CREB-miR-373 signalling axis promoting pancreatic cancer growth, explaining in part the mechanistic insights controlling this phenomenon.

To date, many studies have focused on microRNAs and their regulated targets, but few studies have investigated how microRNA expression is controlled transcriptionally. Deletion of CREB binding sites within the miR-373 promoter abrogated ZIP4-mediated enhancement of expression, demonstrating a direct role for CREB in controlling microRNA levels. This data suggest that ZIP4-dependent CREB activation is an important event in regulating miR-373 transcription in pancreatic cancer cells and possibly other cancer types. In addition, this work provides a novel paradigm defining how dysregulated expression of a zinc importer has phenotypic consequences in microRNA regulation.

MicroRNA expression signatures may have better diagnostic and prognostic value than mRNA profiling in cancer patients (Lu et al, 2005). More importantly, specific microRNAs act as oncogenes or tumour suppressors, promoting cancer initiation, progression, and metastasis. miR-373 has been implicated as a potential ‘onco-miR’ in testicular germ-cell tumours as well as oesophageal, prostate and breast cancers (Huang et al, 2008; Voorhoeve et al, 2006). Recently, miR-373 has been shown to be involved in multiple cancer pathways such as hypoxia response and DNA damage repair, potentially contributing to genomic instability. Furthermore, miR-373 regulates MMP activity via the Ras/Raf/MEK/Erk signalling pathway, increasing cell growth and migration in human fibrosarcoma HT1080 cells (Liu & Wilson, 2012). Our microRNA array data indicate that miR-372 and miR-373 in the miR-371/372/373 cluster were both positively correlated with ZIP4 levels in each of the pairwise comparisons, while miR-150 and miR-200 were inversely correlated (Fig 1A). The miR-371/372/373 cluster was selected for further study as the role of miR-373 in pancreatic cancer was unknown and it was among the most upregulated miRNAs in our pairwise comparisons. In addition, we were confident in the quality of the PCR assays as miR-372 and miR-373 had similar expression patterns as would be expected from microRNAs generated from the same polycistron. Of note, the PCR array did not include an assay for miR-371. Published deep sequencing data has shown that miR-373 is the predominant microRNA expressed from the miR-371/372/373 cluster (Griffiths-Jones et al, 2008; Kozomara & Griffiths-Jones, 2011). In addition, miR-372 and miR-373 have identical seed regions and are predicted to regulate a nearly identical set of target genes. Therefore, we selected miR-373 for further investigation. Here, we demonstrate using gain- and loss-of-function approaches that miR-373 mediates the observed ZIP4-enhancement of pancreatic cancer growth and invasion both *in vitro* and *in vivo*, suggesting that ZIP4-upregulated miR-373 is an important signalling pathway in pancreatic cancer. Taken together, these studies revealed that miR-373 is an oncogenic microRNA in pancreatic cancer, promoting growth and metastasis.

Since miR-373 is overexpressed in ZIP4-upregulated pancreatic cancer cells and microRNAs function as negative regulators of gene expression, we hypothesise that miR-373 may repress key tumour suppressors in pancreatic cancer. Although targets for miR-373 have been investigated in other cancer types, this study provides the first description of miR-373 regulated genes in pancreatic cancer. These findings are significant, because the effects of specific microRNAs even on the same target gene are often dependent on cellular contexts relating to the complex interplay between alternative RNA processing, RNA secondary structure, and RNA binding proteins that modulate miRNA:mRNA interactions. We found that miR-373 negatively regulates the expression of TP53INP1, LATS2 and CD44 through direct interactions with the 3′UTR of the corresponding genes. Each gene was further defined as a *bona fide* tumour suppressor in pancreatic cancer using both *in vitro* and *in vivo* mouse xenograft experiments. TP53INP1 is a pro-apoptotic stress-induced p53 target gene and restoration of TP53INP1 expression inhibited tumour growth in nude mice (Gironella et al, 2007). LATS2 is an aserine-threonine kinase, and a tumour suppressor that plays an important role in p53 mediated CDK inhibition (Voorhoeve et al, 2006). CD44 is a cell surface receptor for hyaluronan, and a known metastasis suppressor in breast, prostate and colon cancer (Huang et al, 2008; Jaeger et al, 2001; Pereira et al, 2001). These data define a novel signalling cascade initiated by ZIP4 leading to the silencing TP53INP1, LATS2 and CD44 and increase pancreatic cancer growth. These results provide mechanistic insights explaining in part how a zinc transporter modulates cancer cell growth. It also underscores that this newly identified pathway may act in concert with other signalling cascades sharing CREB as a transcriptional effector (Zhang et al, 2010a).

## MATERIALS AND METHODS

### Human tissue samples

Human pancreatic adenocarcinoma specimens were collected from patients who underwent surgery and were given informed consent according to an IRB-approved human protocol and in accordance with NIH guidelines. All PDAC samples investigated for this study were stage IV.

**The paper explained**

PROBLEM:

Changes in the intracellular levels of the essential micronutrient zinc have been implicated in multiple diseases including pancreatic cancer; however, the molecular mechanism is poorly understood.

RESULTS:

Here we report a novel mechanism where increased zinc mediated by the zinc importer ZIP4 transcriptionally induces miR-373 in pancreatic cancer to promote tumour growth. Our results demonstrate that ZIP4 activates the zinc-dependent transcription factor CREB and requires this transcription factor to increase miR-373 expression through the regulation of its promoter. miR-373 induction is necessary for efficient ZIP4-dependent enhancement of cell proliferation, invasion, and tumour growth. Further analysis of miR-373 *in vivo* oncogenic function reveals that it is mediated at least in part through its negative regulation of TP53INP1, LATS2, and CD44, molecules with known anti-tumoural function.

IMPACT:

Our findings define a novel ZIP4-CREB-miR-373 signalling axis promoting pancreatic cancer growth, providing potential mechanistic insights on how a zinc transporter functions in cancer cells and may have broader implications as inappropriate regulation of intracellular zinc levels plays an important role in many other diseases.

### Chemicals and cell culture

Human pancreatic cancer cell lines MIA PaCa-2, AsPC-1 and BxPC-3 were purchased from the American Type Culture Collection (ATCC, Rockville, MD), and were cultured as previously described (Li et al, 2007). The human ZIP4 antibody was generated in rabbits against a KLH-conjugated 14-aa synthetic peptide as previously described (Liuzzi et al, 2004). Other chemicals were purchased from Sigma (St. Louis, MO).

### Stable cell line selection

AsPC-shZIP4 cells were infected with the lentivector-based miRNA precursor constructs (System Biosciences Cat# PMIRH373PA-1) for miR-373 overexpression, and stable cells were selected (AsPC-shZIP4-Pre373). MIA-ZIP4 cells were infected with the miRZip anti-microRNAs lentiviral-based miRNA knockdown vector (System Biosciences) expressing anti-sense miR-373, and stable cells were selected (MIA-ZIP4-Anti373). Three individual clones were selected for each stable cell. To investigate the functional involvement of the miR-373 target genes TP53INP1, LATS2 and CD44, shRNA retrovirus vectors (Origene, Rockville, MD) were used to select stable knockdown cells in MIA PaCa-2 cells (Li et al, 2009).

### RNA extraction and real time PCR

Total microRNAs were extracted using the Ambion mirVana miRNA isolation kit (Life Technologies), and the microRNAs were reverse transcribed with the QuantiMir RT kit (SBI). Real time PCR was performed to detect the miRNA expression (Zhang et al, 2009). The β-actin and U6 snRNA were used as internal controls for gene and miRNA expression. For PCR array analysis, the differential expression of 95 miRNAs in ZIP4 overexpressed or silenced cells (MIA-ZIP4 and AsPC-shZIP4) and xenografts was analysed by RT-PCR using QuantiMir System (SBI System Biosciences). The 95 miRNAs selected for the array are based on their potential roles in cancer. The array plate was pre-coated with the RT-PCR primers of the 95 miRNAs and the U6 transcript was included as a normalisation control. The cDNAs from different cell lines and tissues were mixed with SYBR® Green Mastermix (Bio-Rad Laboratories, Hercules, CA) plus the universal reverse primer. The fold change of each miRNA was calculated and standardised based on the U6 level, and was shown as the ratio of the miRNA expression levels in MIA-ZIP4 versus MIA-V group. The expressions of miRNAs in the pancreatic cancer cell and tissue samples were compared by using Student's *t*-test. Data are presented as means of the samples ± SD.

### Western blot analysis and immunohistochemical staining

Cells were lysed and loaded on a 15% SDS-polyacrylamide gel. The membrane was probed with anti-TP53INP1 (1:500), anti-LATS2 (1:500), anti-CD44 (1:1000), or anti-β-actin (1:3000) antibody and detected by using enhanced chemiluminescent (ECL) plus reagent kit. Mouse orthotopic tumours were processed and stained with H&E or Ki67 (Biodesign International) as previously described (Li et al, 2007).

### Chromatin Immunoprecipitation assay (ChIP)

ChIP assay was performed with pancreatic cancer cell line AsPC-1 by using the anti-CREB antibody (Cell Signaling, #9197) and anti-phosphorylated-CREB (Cell Signaling, #9198) with the SimpleChIP Enzymatic Chromatin IP Kit from Cell Signaling (#9003), following the standard protocol.

### Cell proliferation assay

Cell proliferation was analysed with the MTT assay. Stable MIA PaCa-2, BxPC-3 or AsPC-1 cells were seeded in 96-well plates (2 × 10^3^ cells/well), and serum-starved for 24 h. Cell growth was assessed on 0, 1, 2, 3, 4 days after serum starvation. Absorbance was recorded at 490 nm with an EL-800 universal microplate reader (Bio-TekInstruments, Winooski, VT).

### *In vitro* migration and invasion assays

The cell migration/invasion was determined using a modified Boyden chamber assay (Li et al, 2009). Cells were pretreated with 10 μg/ml mitomycin C (sigma, M0503) for 2 h and then cells (10^5^/200 μl) were seeded into the upper compartment of an invasion (Matrigel coated) or migration (uncoated) chamber. The migration/invasion rate was presented as the ratio of the mean fluorescence reading after scraping of the cells divided by the reading before removing the top cells. A monolayer wound-healing assay was also performed. After cells were pretreated with mitomycin C as described, wounds were created in confluent monolayer cells, and wound-healing was observed at 0, 24, 48 and 72 h within the scrape line, and representative fields at different time points were photographed.

### Pancreatic cancer xenograft mouse model

Sub-confluent stable cells lines (3 × 10^6^) were inoculated either into the right flank (subcutaneous tumour model) or the body of the pancreas (orthotopic tumour model) of 6- to 8-week-old male nude mice (NCI-Charles River, MD) (Li et al, 2009). All mice were cared for in accordance with the OPRR and Animal Welfare Act guidelines. After 4 weeks, all surviving mice were euthanised and evaluated macroscopically for the presence of orthotopic tumours and the metastases in the abdominal cavity. The tissue sections were stained with H&E, and the microscopic images were analysed with NIH ImageJ to define the area of the tumour cells (tumour area) and the surrounding cells (none tumour area). Tumour area is defined as the percentage of tumour cells versus surrounding benign cells such as stromal, duct, and acinar cells (Jeong et al, 2005; Saruc et al, 2010).

### 3′ UTR analysis of miR-373 target genes

The luciferase reporter vector containing the 3′UTR regions of TP53INP1, LATS2, and CD44 were purchased from GeneCopoeia. The miR-373 binding sites were mutated using the QuikChange II XL Site-Directed Mutagenesis Kit (Agilent). The wild type or mutant 3'UTR reporter vectors were co-transfected into pancreatic cancer or HEK293 cells together with the plasmid that overexpresses miR-373 (Pre373) or control vector (PreC). The reporter gene activity was determined by measuring the luciferase activity using Dual-Luciferase®Reporter Assay System (Promega, WI). The luciferase activity was normalised with the activity of the internal control enzyme, Renilla luciferase on the same vector, to adjust the transfection efficiency between different wells.

### Promoter activity Assay

The 770 bp (short) and 2.5 kb (long) promoter regions of miR-373 were cloned into pGL4.10-basic reporter vector, and the mutations of predicted CREB binding sites were made. The miR-373 promoter reporter vectors were cotransfected with control plasmid pRL-TK into AsPC-1 cells, and the promoter activity was determined by a luciferase assay.

### Statistical analysis

Quantitative results are shown as means ± SD. The statistical analysis was performed by Student's *t* test or Wilcoxon rank sum test between control and treatment groups. Analysis based on the Pearson correlation or Kendall tau rank correlation coefficient was employed to analyse the expression data of miR-373 and ZIP4 in human pancreatic cancer tissue pairs. A *p*-value of <0.05 was considered statistically significant.

## Supplementary material

As a service to our authors and readers, this journal provides supporting information supplied by the authors. Such materials are peer reviewed and may be re-organised for online delivery, but are not copy-edited or typeset. Technical support issues arising from supporting information (other than missing files) should be addressed to the authors.
