# Supplementary material for: A novel epigenetic CREB-miR-373 axis mediates ZIP4-induced pancreatic cancer growth
Source: EMBO Mol Med. 2013 Jul 16;5(9):1322–34. doi: 10.1002/emmm.201302507 (PMC3799489; doi:10.1002/emmm.201302507)
Supplement: Supplementary file 1 [file emmm0005-1322-SD1.pdf]

EMBO Molecular Medicine

A Novel Epigenetic CREB-miR-373 Axis Mediates ZIP4-Induced Pancreatic Cancer Growth

Yuqing Zhang, Jingxuan Yang, Xiaobo Cui, Yong Chen, Vivian F. Zhu, John P. Hagan, Huamin Wang, Xianjun Yu, Sally E. Hodges, Jing Fang, Paul J. Chiao, Craig D. Logsdon, William E. Fisher, F. Charles Brunicardi, Changyi Chen, Qizhi Yao, Martin E. Fernandez-Zapico, and Min Li

Corresponding author: Min Li, The University of Texas Medical School at Houston

Review timeline:

Submission date:	15 January 2013
Editorial Decision:	19 February 2013
Revision received:	13 May 2013
Editorial Decision:	27 May 2013
Revision received:	04 June 2013
Editorial Decision:	06 June 2013
Revision received:	10 June 2013
Accepted:	11 June 2013

Transaction Report:

(Note: With the exception of the correction of typographical or spelling errors that could be a source of ambiguity, letters and reports are not edited. The original formatting of letters and referee reports may not be reflected in this compilation.)

Editor: Roberto Buccione

1st Editorial Decision 19 February 2013

Thank you for the submission of your manuscript to EMBO Molecular Medicine. We have now received comments from the three Reviewers whom we asked to evaluate your manuscript

You will see that while that all three Reviewers are generally supportive of you work, they raise significant issues that question the conclusiveness of the results and note several technical issues that prevent us from considering publication at this time. I will not dwell into much detail, as the evaluations are detailed and self-explanatory. I would like, however, to highlight a few main points.

Reviewer 1 lists a number of specific concerns that need your action. Among these, s/he notes that the ZIP4 overexpression/silencing experiments should be performed on both cell lines and suggests that the decreased cell proliferation observed after miR-373 inhibition should be better analysed to determine the root cause (cell cycle vs. apoptosis). Reviewer 1 also points to a number of flaws in the presentation and discussion of results, which ultimately hamper interpretation. Finally, s/he queries the compatibility of the experiments shown in Fig 6B with current ethical requirements. The latter is an important issue that deserves full clarification.

Reviewer 2 also remarks on a number of issues, the most relevant of which is the insufficient quality of the provided statistical analyses. I should also mention that Reviewers 1 and 3 also point to shortcomings in terms of statistical significance and power for certain experiments. Ultimately, the conclusions are not sufficiently supported by the data as things stand and might require extensive

experimental repetition. Reviewer 2 also notes that additional experiments are required to show whether the Zip4-dependent effects are indeed mediated through the modulation of Zn levels or by other unknown factors. S/he also lists other important points that require your action.

Reviewer 3, similar to Reviewer 1 points to a number of flaws in the presentation and discussion of results and existing literature. I agree that these points are well taken and need to be remedied at both textual and experimental levels.

While publication of the paper cannot be considered at this stage, we would be prepared to consider a suitably revised submission, with the understanding that the Reviewers' concerns must be fully addressed with additional experimental data where appropriate and that acceptance of the manuscript will entail a second round of review.

Since the revision in this case appears to require a significant amount of time, additional work and experimentation, I would therefore understand if you chose to rather seek publication elsewhere at this stage. Should you do so, we would welcome a message to this effect.
Please note that it is EMBO Molecular Medicine policy to allow a single round of revision only and that, therefore, acceptance or rejection of the manuscript will depend on the completeness of your responses included in the next, final version of the manuscript.

As you know, EMBO Molecular Medicine has a "scooping protection" policy, whereby similar findings that are published by others during review or revision are not a criterion for rejection. However, I do ask you to get in touch with us after three months if you have not completed your revision, to update us on the status. Please also contact us as soon as possible if similar work is published elsewhere.

***** Reviewer's comments *****

Referee #1 (Comments on Novelty/Model System):

The manuscript reports novel findings which can have a significant impact in the understanding on the molecular mechanisms underlying pancreatic tumor development. This is a tumor wit a rather poor therapeutic treatment success due to the lack of fundamental mechanistic molecular events.

Referee #1 (General Remarks):

In this manuscript, the authors show that ZIP4 induces the expression of miR-373 in pancreatic cancer cells by activating CREB. Moreover, they show that TP53INP1, LATS2 and CD44 are miR-373 targets, involved in tumorigenesis.
Major comments:
- In Fig. 1A, miRNA expression profiling is shown. The authors should include the methodology used for statistical analysis of the data in the material and methods section.
- In all experiments ZIP4 has been overexpressed in MIA cells and silenced in ASPC cells. The authors should show the levels of ZIP4 in both cell lines. Moreover they should explain why overexpression and silencing have not been performed in both cell lines. The results obtained by silencing ZIP4 in ASPC should be confirmed in MIA cells and vice versa.
- In Fig.2C and S2A, the authors show luciferase assays using "pGL 4.10+770bp" and "pGL 4.10+2.5Kb". They should explain what "+770" and "+2.5Kb" mean.
- Fig.2D. The authors state that "Deletion of predicted CREB binding sites #2 and #6 (Mut-2 and Mut-5) within the miR-373 promoter region reduced ZIP4-induced luciferase activity in pancreatic cancer cells". Aren't Mut-1 and Mut-3 statistically relevant in pancreatic cancer cells as well?
- In Fig.3C the authors show that inhibition of miR-373 expression leads to a decrease in cell proliferation. However, they do not provide an explanation for this phenomenon. Are they observing cell death or cell cycle arrest? Cell cycle distribution should be evaluated by flow cytometry. BrdU assay (to determine the percentage of cells in S phase) and Annexin V (to investigate apoptosis) should be performed as well. TUNEL assay is discouraged as miR-373 is involved in DNA damage response.
- In Fig.4, the authors injected TP53INP1, LATS2 and CD44 silenced MIA-PacCa-2 cells both subcutaneously and orthotopically and analyzed the tumor growth in this two systems and found that

silencing of these proteins significantly increased the tumor growth and metastatization. By comparing the histological analysis of silenced tumors with the control ones, authors assessed that the percentage of the tumor area is higher in the former. Could the authors explain what they mean by "tumor area"? (Do they refer to the percentage of growing MIA-PaCa-2 cells respect to the stromal component?)
- The sentence "Those results indicate that repressing of TP53INP1, LATS2 and CD44 led to a decrease tumor growth [...]" (end of results section) is in contrast with the previous analysis of the in vivo models.
- In Fig.5D, Pre-373+LATS2m1 should be included.
- In order to corroborate the data shown in Fig.7, percentage of positive Ki67 nuclei should be shown.
- Supplementary Fig.S1A shows Pearson correlation between ZIP4 and miR-373 expression. There is no mention on how expression was measured and no units are indicated on the graph. If ZIP4 expression was assessed by Immunohistochemical staining, sample images of staining intensities should be provided.
- Supplementary Fig.S4E: if possible a more clear-cut WB for CD44 should be provided.
- Supplementary Fig.S6A: data are difficult to interpret and densitometry should be performed.
Minor comments:
- Labeling of the y-axis in Fig. 2F should be consistent with graphs throughout the manuscript. "Relative copy number" should be changed into "Relative miR-373 levels".
- Labeling should be checked in Fig. 5B. "Pre373" should be "Anti-373".
- It would be best to keep omegeneity in Fig.6A: why are 6 days of treatment shown for MIA-shTP53INP1 cells and 4 for the other two? As for shLATS2 and shCD44, cell number appears decreased for MIA-shTP53INP1 cells at 4 days as well.
- Regarding to the tumor sizes showed in fig. 6B, are the MIA-shCD44 tumor sizes compatible with the current ethical restrictions?

Referee #2 (Comments on Novelty/Model System):

As described in my comments to the authors, needed statistical analyses are missing in several places. Novelty and medical impact are high because the authors map out a new regulatory circuit whereby a zinc transporter controls pancreatic cancer cell tumorigenesis.

Referee #2 (General Remarks):

This study maps out an interesting regulatory pathway from Zip4, zinc, CREB, mi-R323 and several of its target genes to pancreatic cancer cell proliferation, migration, and tumorigenesis. Most of the pieces of this puzzle are well supported and the work, overall, is clearly presented. However, a few issues need to be resolved. My main concern is that much of the results lack sufficient replication and/or statistical analysis so many of the conclusions are not well justified based on the evidence presented.

One weak link in the pathway is the role of zinc as an intermediate signaling molecule. The authors should test whether Zip4 overexpression and knockdown alter cytosolic zinc levels as they hypothesize. Zinc-sensing FRET probes like those from Amy Palmer's lab at U Colorado-Boulder (Proc Natl Acad Sci U S A. 108(18):7351-6.) are ideal for this but metallothionein gene expression or MTF-1 reporter assays can also be used as indirect assays. Also, do zinc chelators prevent the effects of Zip4 overexpression? One could titrate in a zinc chelator (e.g. TPEN) into the medium to determine if there is a concentration of chelator that inhibits Zip4-dependent growth in vitro without inhibiting growth of control cells. These data would help establish if the effect of Zip4 is thru zinc levels or via some other and less expected mechanism.

Figure 2A. The effects of Zip4 on CREB phosphorylation are very subtle. This experiment should be replicated multiple times, quantitated normalized to total CREB, and the increase/decrease in phosphorylation reported with statistical analysis.

Figures 2D and S2B. Mut-5 is referred to in the Results section as having statistically significant effects but it is not noted as such in the figures.

Also relevant to Figure 2, the authors present evidence that basal miR-373 expression requires CREB but not whether Zip4-dependent induction of miR-373 requires CREB. Does pGL4.10+2.5kb increase in a Zip4 overexpressing cell and is this increase dependent on CREB sites and inhibited by siCREB?

Figure 3C and 3D require MIA-V-AntiC and MIA-V-Anti373 controls to confirm that decreased miR-373 is not having a general growth/migration inhibition effect independent of Zip4.

Figure 4C. The Results (page 6, line 4) refer to 20% of MIA-ZIP4-Anti373 mice being tumor free but it looks like only one of the five have a GFP-tagged tumor (therefore, 80% are tumor free). In the legend, these mice are referred to as "representative" which is misleading because it suggests that more were examined. However, with n=5 in each group, this is all there were.

Figure 4D, E. The Results section describe these observations as clear cut but fails to note that they are not statistically significant ($p > 0.05$) and therefore these trends must be described much more conservatively. Alternatively, more replicates could be done to shore up the data.

Figure 5A and B. The immunoblot results should be verified with multiple replicates and quantitated for statistical analysis of the differences observed. Some of the effects shown are fairly subtle and require more rigorous analysis. This is also a problem for the results presented in Figure S4. Figure 5B is mislabeled and should read MIA-Anti373 rather than Pre373.

Effects of siRNA on TP53INP1 are not clear from the immunoblots in Figure S6. Was knockdown actually achieved? If not, the results with shTP53INP1 are open to other interpretations.

Figure 7A. Are the differences in these phenotypes statistically significant? If not, more replicates should be added or the data removed.

Figure S1A. The legend refers to $p < 0.01$ while it is stated as $p<0.001$ in the Results section on page 4.

Figure S3D. Are these effects statistically significant? If not, reference to these data should be deleted.

Abstract. Rather than saying that "Zip4 transcriptionally induces miR-373", I would say that "increased zinc levels mediated by Zip4 activity transcriptionally induces miR-373". The current text implies that Zip4 affects transcription directly.

Referee #3 (Comments on Novelty/Model System):

The figure legends are not self-explanatory.
The scale bars for photographs are missing (figure 3D, 7B and various supplementary figures) or are poor (Figure 6B).
The molecular weights and appropriate band-width display (at least 20 Kd on either side) along with densitometric quantification are missing (Figures 2A, 2E, 5A, 5B and various supplementary figures).
The statistical display is poor. At most 5 to 12 mice have been used per group. It is highly unlikely that this small sample size has a normal distribution (as evidenced by figure 4C). Therefore data in Figures should be for all individual mice per group with median and IQR should be depicted/ plotted. Appropriate statistical tests will then have to be used.

Referee #3 (General Remarks):

The authors remain sole and main proponents of ZIP4 upregulation in pancreatic cancer. This is their strength. In this manuscript they propose a novel a novel ZIP4-CREB-miR-373 signaling axis.

However, it remains perplexing that the alternative hypothesis for ZIP4 upregulation and subsequent downstream mechanism proposed by authors in 2010 (Clin Cancer Res. 2010 Mar 1;16(5):1423-30) remains uncited, undiscussed and in direct conflict with current work, despite using similar models (MIA-ZIP4 cells, ASPC-shZIP4, cell lines and xenografts). The abstract of results the previous work is cited here: 'Cyclin D1 was significantly increased in the ZIP4 overexpressing MIA PaCa-2 cells (MIA-ZIP4)-injected orthotopic xenografts and was downregulated in the ZIP4-silenced ASPC-1 (ASPC-shZIP4) group. The phosphorylation of STAT3, an upstream activator of cyclin D1, was increased in MIA-ZIP4 cells and decreased in ASPC-shZIP4 cells. IL-6, a known upstream activator for STAT3, was also found to be significantly increased in the MIA-ZIP4 cells and xenografts and decreased in the ASPC-shZIP4 group. Overexpression of ZIP4 led to a 75% increase of IL-6 promoter activity and caused increased phosphorylation of CREB.'

Few statements are unsupported by either new data or previous work (authors or others);

1. Further analysis identifies the transcription factor cAMP response element-binding protein (CREB) as a mediator of this phenomenon (results for Figure 1-2).
2. increased cell proliferation as indicated by the strong staining of Ki67, compared with that of the MIAV (results for Figure 7).
3. Our results suggest that ZIP4/miR-373 pathway can serve as a new therapeutic target for pancreatic cancer (discussion: the authors have not therapeutically targeted them by drug /small molecule).
4. Lack of discussion of their work in Curr Mol Med 2013 and 2012.
5. Lack of discussion of work in prostate cancer, HCC, breast cancer
6. Lack of discussion of work from their group in Cnacer Biol Ther 2010 with yet another alternative mechanistic hypothesis.

Few experiments require further explanation:
1. How did authors compensate for decrease in proliferation upon migration /invasion effects seen in Figure 3 after knocking down miR373.
2. How many mice had no tumour at all as shown in Figure 4C.
3. What are authors measuring inSupplementary Figure 1A. mRNA, protein. How can they be sure it is only tumour cells (predominant stroma in pancreatic cancer). What is grade IV: do they mean Stage IV. This correlation plot is skewed by two outliers. How did they compensate for this statistically or biologically?
4. What is percentage Ki67 in Figure 7B?
5. How was the data in Figure 7A assessed statistically and accounting for small sample size for the biological relevance?

Furthermore:
1. The figure legends are not self-explanatory.
2. The scale bars for photographs are missing (figure 3D, 7B and various supplementary figures) or are poor (Figure 6B).
3. The molecular weights and appropriate band-width display (at least 20 Kd on either side) along with densitometric quantification are missing (Figures 2A, 2E, 5A, 5B and various supplementary figures).
4. The statistical display is poor. At most 5 to 12 mice have been used per group. It is highly unlikely that this small sample size has a normal distribution (as evidenced by figure 4C). Therefore data in Figures should be for all individual mice per group with median and IQR should be depicted/ plotted. Appropriate statistical tests will then have to be used.

1st Revision - authors' response 13 May 2013

Referee #1

1. In Fig. 1A, miRNA expression profiling is shown. The authors should include the methodology used for statistical analysis of the data in the material and methods section.

Response: We have included the methodology used for the statistical analysis of the miRNA profiling data in the materials & methods section as suggested by the reviewer. The expressions of

miRNAs in the MIA-V, MIA-ZIP4, AsPC-shV and AsPC-shZIP4 pancreatic cancer cells and tissue samples were analyzed with Student's t test. Data are presented as means of samples ± standard deviations. One of our coauthors Dr. Yong Chen, is an experienced statistician at UT School of Public Health, he has carefully revised the statistical sections throughout the manuscript and confirmed our analysis in this study.

2. In all experiments ZIP4 has been overexpressed in MIA cells and silenced in ASPC cells. The authors should show the levels of ZIP4 in both cell lines. Moreover they should explain why overexpression and silencing have not been performed in both cell lines. The results obtained by silencing ZIP4 in ASPC should be confirmed in MIA cells and vice versa.

Response: MIA PaCa-2 cells have the lowest endogenous level of ZIP4 in a panel of 8 pancreatic cancer cells (Panc-1, MIA PaCa-2, BxPC-3, Hs766T, AsPC-1, Capan-1, HPAF-II, and PL45 cell) we examined, and AsPC-1 cells have the highest endogenous ZIP4, that's why we chose MIA PaCa-2 cells to overexpress ZIP4, and chose AsPC-1 cells to silence ZIP4.

In the revised manuscript, we also checked the miR-373 expression in AsPC-1 cells transfected with ZIP4 cDNA, and in MIA PaCa-2 cells transfected with ZIP4 siRNA by Q-PCR to rule out the possible variations between different cells. Our results, similar to what we had in Fig. 1, shows an upregulation of miR-373 in AsPC-1 cells overexpressing ZIP4; conversely silencing of ZIP4 in MIA-PaCa2 cells reduces the expression of miR-373. These new data have been included to the Supporting Information figures as new Fig. S1A and S1B.

3. In Fig.2C and S2A, the authors show luciferase assays using "pGL 4.10+770bp" and "pGL 4.10+2.5Kb". They should explain what "+770" and "+2.5Kb" mean.

Response: We apologize for the confusion. The "+770" and "+2.5kb" labels indicated a 770 bp short fragment and the 2.5kb full length long fragment upstream of the transcriptional start site of miR-373, respectivley. To avoid any confusion, we have relabeled the constructs as "short" and "long" and included in the methods section a description of the insert contained in these constructs.

4. Fig.2D. The authors state that "Deletion of predicted CREB binding sites #2 and #6 (Mut-2 and Mut-5) within the miR-373 promoter region reduced ZIP4-induced luciferase activity in pancreatic cancer cells". Aren't Mut-1 and Mut-3 statistically relevant in pancreatic cancer cells as well?

Response: The decision to focus on Mut-2 and Mut-5 was based on two experimental results: 1) these mutants show the most profound effect in all the analyzed cell lines (MIA PaCa-2, AsPC-1, PL45 and HEK 293 cells) and 2) the effect of Mut-1 and Mut-3 sites was variable throughout all cell lines, especially in the pancreatic cancer cells, in some cases it did not show statistical differences with the control group (Supporting Information Fig. S2E, S2F and S2G). Therefore, we have decided to focus our studies on Mut-2 and Mut-5 as the CREB-responsive elements of this promoter.

5. In Fig.3C the authors show that inhibition of miR-373 expression leads to a decrease in cell proliferation. However, they do not provide an explanation for this phenomenon. Are they observing cell death or cell cycle arrest? Cell cycle distribution should be evaluated by flow cytometry. BrdU assay (to determine the percentage of cells in S phase) and Annexin V (to investigate apoptosis) should be performed as well. TUNEL assay is discouraged as miR-373 is involved in DNA damage response.

Response: As suggested by the reviewer, we have performed cell cycle analysis and determined the levels of apoptosis in MIA-ZIP4-Anti373 and MIA-ZIP4-AntiC cells by flow cytometry using BrdU and Annexin V staining. Our data indicate that blockade of miR-373 caused cell cycle arrest at G0/G1 phase without significant change in the apoptotic rate. The new data has been added in Supporting Information Fig. S3 as new Fig. S3A, S3B and S3C.

6. In Fig.4, the authors injected TP53INP1, LATS2 and CD44 silenced MIA-PaCa-2 cells both subcutaneously and orthotopically and analyzed the tumor growth in this two systems and found that silencing of these proteins significantly increased the tumor growth and metastatization. By comparing the histological analysis of silenced tumors with the control ones, authors assessed that the percentage of the tumor area is higher in the former. Could the authors explain what they mean by "tumor area"? (Do they refer to the percentage of growing MIA-PaCa-2 cells respect to the stromal component?)

Response: Tumor area is defined as the percentage of tumor cells versus surrounding benign cells such as stromal, duct, and acinar cells (Histol Histopathol 25, 423-432 (2010) and World J Gastroenterol 2005;11(43):6765-6769). The tissue sections were stained with H&E, and the microscopic images were analyzed with NIH ImageJ to define the area of the tumor cells (tumor area) and the surrounding cells (none tumor area) (Fig. S4).

7. "Those results indicate that repressing of TP53INP1, LATS2 and CD44 led to a decrease tumor growth [...]" (end of results section) is in contrast with the previous analysis of the in vivo models.

Response: We apologize for the typo, it should say "led to an increased tumor growth". We have corrected it in the revised text.

8. In Fig.5D, Pre-373+LATS2m1 should be included.

Response: We have included the Pre-373+LATS2m1 construct, which showed partial rescue of the luciferase activity, similar as that of Pre-373+LATS2m2. The double mutation Pre-373+LATS2m1m2 completely rescued the luciferase activity repressed by Pre-373. The new data has been included in Fig. 5D.

9. In order to corroborate the data shown in Fig.7, percentage of positive Ki67 nuclei should be shown.

Response: We have added the quantitative data of Ki67 staining in Fig. 7, which indicates that silencing of the miR-373 target genes led to increased cell proliferation in the xenograft tumors. The Ki67 positive cells are 52% (shTP53INP1), 47% (shLATS2), and 65% (shCD44), respectively, compared with the 14% positive cells in the control group (shV).

10. Supporting Information Fig.S1A shows Pearson correlation between ZIP4 and miR-373 expression. There is no mention on how expression was measured and no units are indicated on the graph. If ZIP4 expression was assessed by Immunohistochemical staining, sample images of staining intensities should be provided.

Response: The expression of ZIP4 and miR-373 were assessed by Q-PCR. We have added the explanation and expression units in Supporting Information Fig. S1.

11. Supporting Information Fig.S4E: if possible a more clear-cut WB for CD44 should be provided.

Response: We have replaced the previously submitted blot with a new clear-cut WB picture for CD44, which indicates that blocking CREB by siRNA caused a significant increase of CD44 in MIA PaCa-2 cells.

12. Supporting Information Fig.S6A: data are difficult to interpret and densitometry should be performed.

Response: We have repeated the immunoblot at least three times, and included more clear-cut blots. We also added the densitometry data in Fig. S6A.

© EMBO

13. Labeling of the y-axis in Fig. 2F should be consistent with graphs throughout the manuscript. "Relative copy number" should be changed into "Relative miR-373 levels".

Response: We have changed to "Relative miR-373 levels" in Fig. 2F as suggested by the reviewer.

14. Labeling should be checked in Fig. 5B. "Pre373" should be "Anti-373".

Response: We apologize for the typo, and have corrected the labeling to Anti373 in Fig. 5B.

15. It would be best to keep homogeneity in Fig.6A: why are 6 days of treatment shown for MIA-shTP53INP1 cells and 4 for the other two? As for shLATS2 and shCD44, cell number appears decreased for MIA-shTP53INP1 cells at 4 days as well.

Response: As suggested by the reviewer, we have revised the Fig. 6A, and show only MTT data up to 4 days for all three miR-373 target genes. As shown in the revised manuscript, individual silencing of miR-373 targeted genes (TP53INP1, LATS2 and CD44) increased cell proliferation compared to the control cells (MIA-shV) after 4 days culture.

16. Regarding to the tumor sizes showed in fig. 6B, are the MIA-shCD44 tumor sizes compatible with the current ethical restrictions?

Response: We thank the reviewer for this critical comment. Yes, the tumor size is compatible with the ethical restrictions and was approved by the Animal Welfare Committee (AWC) at the University of Texas Health Science Center (UTHSC). In this experiment, there are 10 ~ 12 mice for each group orthotopically injected with human pancreatic cancer cell lines with target genes stably silenced. Mice were closely observed daily till 4 weeks. Analgesia was provided whenever appropriate. Then mice were euthanized and the xenograft tumors were collected for pathological analysis. These are orthotopic tumors in mice pancreas (not subcutaneous tumors), and the tumor weight is less than 10% of the mouse body weight, which are acceptable by Institutional Animal Care Use Committee at UTHSC.

Referee #2

1. One weak link in the pathway is the role of zinc as an intermediate signaling molecule. do zinc chelators prevent the effects of Zip4 overexpression? One could titrate in a zinc chelator (e.g. TPEN) into the medium to determine if there is a concentration of chelator that inhibits Zip4-dependent growth in vitro without inhibiting growth of control cells. These data would help establish if the effect of Zip4 is thru zinc levels or via some other and less expected mechanism.

Response: As suggested by the reviewer, we have performed additional experiments in both MIA PaCa-2 and AsPC-1 cells using TPEN treatment. We depleted the intracellular zinc with this chelator, and added dose-dependent $ZnCl_2$ back into the culture medium, and we found that ZIP4 promotes zinc-dependent upregulation of miR-373 in a narrow range, and this range of concentration is different between MIA PaCa-2 and AsPC-1 cells. When zinc concentration is low (low zinc, 1uM), ZIP4 overexpression caused increased miR-373 in MIA PaCa-2 cells (MIA-V vs MIA-ZIP4), but when the zinc concentration reaches a threshold (high zinc, 25 uM), ZIP4 overexpression leads to decreased miR-373 (MIA-V vs MIA-ZIP4). Conversely, ZIP4 silencing causes decreased miR-373 in a zinc-dependent manner in AsPC-1 cells (AsPC-shV vs AsPC-shZIP4), within even smaller concentration range (Low zinc: 0.5uM, high zinc: 10uM). Thus, together these results support a role for zinc as intermediate signaling molecule regulating ZIP4 effects. We have added the new data in Supporting Information Fig. S1 as new Fig. S1F and S1G.

2. Figure 2A. The effects of Zip4 on CREB phosphorylation are very subtle. This experiment should be replicated multiple times, quantitated normalized to total CREB, and the increase/decrease in phosphorylation reported with statistical analysis.

Response: We have repeated the CREB phosphorylation immunoblot three times, and included a more clear-cut blot in Fig. 2, which shows substantial changes in CREB phosphorylation upon ZIP4 overexpression or silencing. We have also added to this figure densitometry and statistical analysis.

3. Figures 2D and S2B. Mut-5 is referred to in the Results section as having statistically significant effects but it is not noted as such in the figures.

Response: We have added an asterisk "*" on Mut5 in Fig. 2D and S2 indicating the statistical significance.

4. Also relevant to Figure 2, the authors present evidence that basal miR-373 expression requires CREB but not whether Zip4-dependent induction of miR-373 requires CREB. Does pGL4.10+2.5kb increase in a Zip4 overexpressing cell and is this increase dependent on CREB sites and inhibited by siCREB?

Response: We have performed additional experiment in both MIA-V and MIA-ZIP4 cells transfected with siCREB. We found that ZIP4 significantly induces miR-373 promoter activity compared with the vector control, and the siCREB blocks ZIP4-dependent activation of the miR-373 promoter. The new data indicate that not just basal miR-373 expression requires CREB, but the ZIP4-dependent induction of miR-373 also requires an active CREB transcription factor (new Supporting Information Fig. S2I).

5. Figure 3C and 3D require MIA-V-AntiC and MIA-V-Anti373 controls to confirm that decreased miR-373 is not having a general growth/migration inhibition effect independent of Zip4.

Response: We have added new experiments to repeat the cell proliferation/migration assay in MIA-V cells transfected with AntiC and Anti373 constructs, which showed unchanged cell proliferation and migration upon miR-373 blocking, indicating that decreased miR-373 is not having a general growth/ migration inhibition effect independent of ZIP4. The new data is included in Supporting Information Fig. S3 as new Fig. S3D and S3E.

6. Figure 4C. The Results (page 6, line 4) refer to 20% of MIA-ZIP4-Anti373 mice being tumor free but it looks like only one of the five have a GFP-tagged tumor (therefore, 80% are tumor free). In the legend, these mice are referred to as "representative" which is misleading because it suggests that more were examined. However, with n=5 in each group, this is all there were.

Response: One of the five mice showed GFP-tagged tumor in MIA-ZIP4-Anti373 group because of the limitation of the imaging instrument to detect the GFP-tagged tumor in mice abdomen, but after euthanization and dissection, four of the five mice showed pancreatic tumors with only one mouse being tumor free. We have revised the figure legend and removed the "representative" in Fig. 4C to better reflect the finding of this experiment.

7. Figure 4D, E. The Results section describe these observations as clear cut but fails to note that they are not statistically significant ($p > 0.05$) and therefore these trends must be described much more conservatively.

Response: We have rephrased the results section as suggested, and pointed out the limitation on statistical significance.

8. Figure 5A and B. The immunoblot results should be verified with multiple replicates and quantitated for statistical analysis of the differences observed. Some of the effects shown are fairly subtle and require more rigorous analysis. This is also a problem for the results presented in Figure S4. Figure 5B is mislabeled and should read MIA-Anti373 rather than Pre373.

Response: We have repeated the immunoblots at least three times, and have added the quantitative data with statistical analysis in Fig. 5A, 5B, and S5. All the replicas show similar results. We have also corrected the figure legend of Fig. 5B following the reviewer's suggestions.

9. Effects of siRNA on TP53INP1 are not clear from the immunoblots in Figure S6. Was knockdown actually achieved? If not, the results with shTP53INP1 are open to other interpretations.

Response: All those cells used here are stable cells lines, which were confirmed with the knocking down of the target gene on both mRNA and protein levels by real time RT-PCR and immunoblotting. We have repeated this experiment multiple times and replaced the TP53INP1 blot with a more clear-cut blot (Fig. S6). We have also added the quantitative densitometry data in Fig. S6A.

10. Figure 7A. Are the differences in these phenotypes statistically significant? If not, more replicates should be added or the data removed.

Response: We re-conducted the analysis using Fisher's exact test. Specifically, we investigated one outcome at a time (i.e., ascites, peritoneal dissemination, liver metastasis, spleen metastasis or colon metastasis) and tested for any difference between the four groups of mices (i.e., MIA-shV, MIA-shLATS2, MIA-shTP53INP1 and MIA-shCD44). Fisher's exact tests on each of the constructed 2x4 tables suggested that there is significant evidence in difference between groups in colon metastasis (P=0.05), marginal evidence in difference between groups in liver metastasis (P=0.11) and spleen metastasis (P=0.16), mild evidence in difference between groups in peritoneal dissemination (P=0.21), and there is no significant difference between groups in ascites (p=0.91). Given the small sample size and the overall marginal evidence, we moved Fig. 7A to the Supporting Information data as the new Fig. S7. We have also revised our statements in the text to point out that although the silencing of TP53INP1, LATS2 and CD44 in MIA PaCa-2 cells significantly increased tumor cell proliferation and primary tumor growth compared with the vector control cells (Fig. 6B and Fig. 7), it may have limited effect in tumor progression except for the amount of colon metastasis (Fig. S7). These data suggest that repressing of TP53INP1, LATS2 and CD44 may be part of the mechanism underlying ZIP4/miR-373 induced pancreatic tumor growth.

11. Fig S1A. The legend refers to $p < 0.01$ while it is stated as $p<0.001$ in the Results section on page 4.

Response: We have revised the statistics of Fig. S1 and updated both the figure legend and the results section. Now they are consistent (P<0.01).

12. Fig S3D. Are these effects statistically significant? If not, reference to these data should be deleted.

Response: We have deleted Figure S3D as suggested.

13. Abstract. Rather than saying that "Zip4 transcriptionally induces miR-373", I would say that "increased zinc levels mediated by Zip4 activity transcriptionally induces miR-373". The current text implies that Zip4 affects transcription directly.

Response: We have rephrased the abstract according to the reviewer's suggestion.

Referee #3

1. The authors remain sole and main proponents of ZIP4 upregulation in pancreatic cancer. This is their strength. In this manuscript they propose a novel ZIP4-CREB-miR-373 signaling axis. However, it remains alternative hypothesis for ZIP4 upregulation and subsequent downstream mechanism proposed by other reports remains uncited, undiscussed and in direct conflict with current work, despite using similar models.

Response: We appreciate the reviewer's comment, and have added the suggested references of these previous studies to the revised manuscript and compared them with our current data in the

discussion section. As mentioned by the reviewer, the role of zinc and zinc transporters in cancer have been studied in breast cancer and HCC. Zinc importers promote cell proliferation, migration, and metastasis. However, they play an inhibitory role in prostate cancer (Golovine, 2008; Makhov 2008; Weaver 2010; Taylor et al, 2003; Kagara et al, 2007). These studies indicate a tissue specific or tumor specific function of zinc transporters in different cancer types. Our group have recently demonstrated a novel biological role for the zinc importer ZIP4 in pancreatic cancer (Li 2009; Li 2007; Yang 2013; Zhang 2010), and have shown that ZIP4 is a major zinc importer upregulated in pancreatic cancer, and promotes cancer growth. However, the detailed mechanism of how ZIP4 overexpression causes the activation of the downstream signaling pathways in pancreatic cancer is not clear. In this study, we report a novel mechanism where increased zinc levels mediated by a zinc importer ZIP4 transcriptionally induces miR-373 in a CREB-dependent manner in pancreatic cancer cells. These results define a novel ZIP4-CREB-miR-373 signaling axis promoting pancreatic cancer growth, providing mechanistic insights on how a zinc transporter functions in cancer cells and may have broader implications as inappropriate regulation of intracellular zinc levels plays an important role in many other diseases with dysregulated zinc levels.

2. Further analysis is needed to identify the role of the transcription factor cAMP response element-binding protein (CREB) as a mediator of this phenomenon.

Response: Using a comibnation of bioinformatics, deletion mutagenesis, reporter, expression, RNAi and chromatin immunoprecipitation assays, we were able to demonstrate an effector role of CREB downstream of ZIP4. Bioinformatics analysis of the promoter region of miR-373 found several binding sites of a zinc-dependent transcription factor CREB (Fig. 2B and Fig. S2B). We have cloned the promoter region of miR-373 into a reporter vector and shown that deletion of some of these CREB binding sites significantly impact the ZIP4-dependent activation of this activity (Fig. 2D, Fig. S2E, S2F, and S2G); Chromatin immunoprecipitation assay confirmed CREB binding to the miR-373 promoter (Fig. 2E); further knocking down of CREB blocks in ZIP4-induced miR-373 expression (Fig. 2F). Collectively, these results support the role of CREB as a functional effector of ZIP4 in pancreatic cancer.

3. The increased cell proliferation as indicated by the strong staining of Ki67, should be compared with that of the MIAV (results for Figure 7).

Response:. The IHC staining data has been quantified, and the new data has been added as a new Fig. 7B. This quantization shows that silencing of the miR-373 target genes caused increased cell proliferation as indicated by strong staining of Ki67, compared with weak staining of Ki67 in control cells.

4. Our results suggest that ZIP4/miR-373 pathway can serve as a new therapeutic target for pancreatic cancer (discussion: the authors have not therapeutically targeted them by drug /small molecule).

Response: Our results indicated that ZIP4/miR-373 signaling pathway plays an important role in pancreatic cancer growth, therefore, we speculate that this pathway might serve as a new potential target for pancreatic cancer therapy. Although we have not shown the therapeutic data, which we believe is beyond the scope of this paper, our unpublished results indicate that silencing ZIP4 by liposome-delivered shRNA inhibits pancreatic cancer growth, and prolongs survival in a mouse model. These exciting data suggest pancreatic cancer growth can be inhibited with direct targeting of specifically over-expressed genes in pancreatic cancer by 3 cycles treatment of iv liposome/shRNA, and demonstrate the feasibility of a novel shRNA therapy targeting ZIP4 in pancreatic cancer. These data was not included in the revised manuscript, however, if the reviewer considers that it should be included we will be happy to do so.

5. Lack of discussion of the work by the authors and others in prostate cancer, HCC, breast cancer.

Response: As suggested by the reviewer, we have included the discussion of our previous work (Curr Mol Med and PNAS), as well as the work in prostate cancer, HCC, and breast cancer.

6. How did authors compensate for decrease in proliferation upon migration /invasion effects seen in Figure 3 after knocking down miR373.

Response: We used cell proliferation inhibitor (mitomycin C) to treat the cells before we did the migration/invasion assay to make sure that the reduced migration/invasion is not due to decreased cell proliferation. Briefly, both cells were incubated with 10 ug/ml mitomycin C for 2 hr and then cells was washed to remove the residual mitomycin C before proceeding to the migration/invasion assay. The results included in Fig. 3D show blocking miR-373 can decrease cellular migration activity, even in the presence of ZIP4 overexpression, suggesting that miR-373 is a mediator of the ZIP4 effect in pancreatic cancer.

7. How many mice had no tumour at all as shown in Figure 4C.

Response: One of the five mice showed GFP-tagged tumor in MIA-ZIP4-Anti373 group because of the limitation of the imaging to detect the GFP-tagged tumor in mice abdomen, but after euthanization and dissection, four of the five mice showed pancreatic tumor with only one mouse being tumor free. The sizes of all the four tumors from MIA-ZIP4-Anti373 group are much smaller than that from MIA-ZIP4-AntiC group, indicating that knocking down of miR-373 in MIA-ZIP4 cells impairs ZIP4 induced tumor growth in vivo. This has been clarified in the revised manuscript.

8. What are authors measuring in Supporting Information Figure 1A. mRNA, protein. How can they be sure it is only tumour cells (predominant stroma in pancreatic cancer).

Response: We measured the mRNA level for ZIP4 and miR-373. We agree that the predominant cells are stroma in pancreatic cancer, but the expression level of ZIP4 is very low is stroma and benign tissues, as shown in the new data of IHC staining of ZIP4 protein with human pancreatic cancer sections included in Supporting Information Fig. S1D and S1E.

9. What is grade IV: do they mean Stage IV.

Response: We apologize for the confusion, we meant stage IV not grade IV.

10. This correlation plot is skewed by two outliers. How did they compensate for this statistically or biologically?

Response: According to the analysis of our expert biostatistician, Dr. Chen, the exclusion of several outliers from cohort 1 (Baylor College of Medicine, Houston, TX, USA) using various cut values (leading to smaller samples, n=15), allow us to still observe positive correlations, although they are less statistically significant due to smaller sample size (Pearson correlation coefficient = 0.670; Kendall's tau rank correlation coefficient = 0.172 based on n=15 samples). To validate the positive correlation, we have expanded the sample size by including more human pancreatic cancer samples from a separate cohort 2 (Shanghai Cancer Center, Shanghai, China, n=25), which also showed similar positive correlations of ZIP4 and miR-373 expression levels (Pearson correlation coefficient = 0.641, P=0.001; Kendall's tau rank correlation coefficient = 0.320, P=0.025). Please see the two new plots as below. We did not pool two sets of data together to avoid the potential confounding effect of study site. We believe that ZIP4 and miR-373 are positively correlated in human pancreatic cancer.

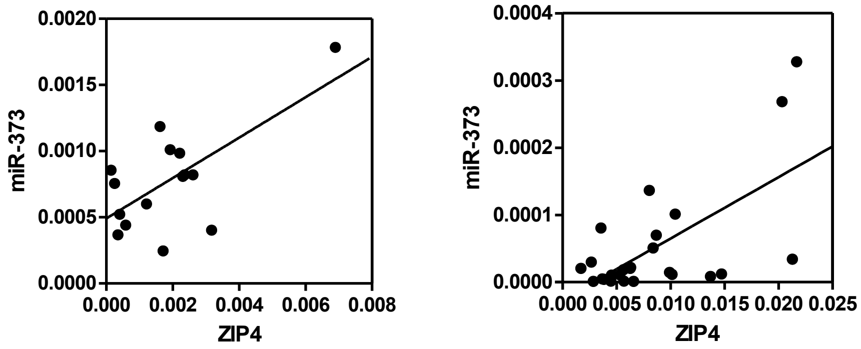

11. What is percentage Ki67 in Figure 7B?

Response: We have added the quantitative data in Fig. 7B, showing that silencing of the miR-373 target genes led to increased cell proliferation in the xenografts. The Ki67 positive cells are 52% (shTP53INP1), 47% (shLATS2), and 65% (shCD44), respectively, compared with the 14% positive cells in the control group (shV).

12. How was the data in Figure 7A assessed statistically and accounting for small sample size for the biological relevance?

Response: Following the reviewer's comment, we re-conducted the analysis using Fisher's exact test. Specifically, we investigated one outcome at a time (i.e., ascites, peritoneal dissemination, liver metastasis, spleen metastasis or colon metastasis) and tested for any difference between the four groups of mices (i.e., MIA-shV, MIA-shLATS2, MIA-shTP53INP1 and MIA-shCD44). Fisher's exact tests on each of the constructed 2x4 tables suggested that there is significant evidence in difference between groups in colon metastasis (P=0.05), marginal evidence in difference between groups in liver metastasis (P=0.11) and spleen metastasis (P=0.16), mild evidence in difference between groups in peritoneal dissemination (P=0.21), and there is no significant difference between groups in ascites (p=0.91). Given the small sample size and the overall marginal evidence, we moved Fig. 7A to the Supporting Information data as the new Fig. S7. We have also revised our statements in the text to point out that although the silencing of TP53INP1, LATS2 and CD44 in MIA PaCa-2 cells significantly increased tumor cell proliferation and primary tumor growth compared with the vector control cells (Fig. 6B and Fig. 7), it may have limited effect in tumor progression except for the amount of colon metastasis (Fig. S7). These data suggest that repressing of TP53INP1, LATS2 and CD44 may be part of the mechanism underlying ZIP4/miR-373 induced pancreatic tumor growth.

13. The figure legends are not self-explanatory.

Response: We apologize for the confusion, and have revised all the figure legends to be more informative as suggested by the reviewer.

14. The scale bars for photographs are missing (figure 3D, 7B and various Supporting Information figures) or are poor (Figure 6B).

Response: We have corrected the scale bars in the above mentioned figures.

15. The molecular weights and appropriate band-width display (at least 20 Kd on either side) along with densitometric quantification are missing (Figures 2A, 2E, 5A, 5B and various Supporting Information figures).

Response: We have added the molecular weights and appropriate band-width display. We have also added densitometry of all immunoblots in the revised figures.

16. It is highly unlikely that this small sample size has a normal distribution (as evidenced by figure 4C). Therefore data in Figures should be for all individual mice per group with median and IQR should be depicted/ plotted. Appropriate statistical tests will then have to be used.

Response: We have revised the figure legend and text, and have reconfirmed the statistical analysis with an experienced statistician Dr. Yong Chen, and validated our results. We have represented the data from Figure 4C with boxplot chart in supplement figure S4D, the statistical analysis result showed that: for the orthotopic tumor data, P=0.01 using two-sided t-test, and P=0.008 using two-sided Wilcoxon rank sum test; for the subcutaneous tumor data, P=0.004 using two-sided t-test, and P=0.008 using two-sided Wilcoxon rank sum test.

2nd Editorial Decision 27 May 2013

Thank you for the submission of your revised manuscript to EMBO Molecular Medicine.
We have now heard back from the three Reviewers whom we asked to re-evaluate your revised manuscript.

You will see that all three Reviewers have remaining issues that prevent us from considering publication at this time.

Reviewer 1, while acknowledging your replies to his/her previous comments, feels that additional clarification is required for Figures 2D, S3A, S3B and S4, implying appropriate amendments in the manuscript. Reviewer 1 also suggests that haematoxylin counterstaining is required in Fig. 7 to better distinguish Ki-67+ vs. Ki-67- nuclei.

Reviewer 2 points to a potential caveat that appears to compromise a tenet of the manuscript, i.e. that Zip4 overexpression triggers increased miR-373 expression by increasing cytosolic zinc. Indeed, s/he notes that the new data presented in Figure S1F contradicts that model, as increased miR-373 expression appears to occur when intracellular zinc is decreased by treatment with TPEN, thus suggesting that Zip4 may be acting in a different fashion to increase miR-373 expression. During Reviewer cross commenting, Reviewer 3 agreed with this concern. I am sure that you will understand that this issue is a critical one and requires full clarification.

Reviewer 3 has some remaining concerns as well. Firstly, s/he would like to see that the statistical analysis in Fig. 4 shown as individual mice data. Reviewer 3 would also like a proper discussion of the potentially misleading claims made in the Abstract and Discussion with respect to your previously published data.

As you know, we would normally not allow a second revision. I am prepared in this case, however, to give you another opportunity to improve your manuscript, with the understanding that the Reviewers' concerns must be fully addressed with additional experimental data where appropriate and that next version of the manuscript will undergo a third round of review.

I look forward to seeing a revised form of your manuscript as soon as possible.

***** Reviewer's comments *****

Referee #1 (General Remarks):

The authors have clarified all the issues raised by this reviewer. Only minor points remain to be clarified.

4. Fig.2D. The authors state that "Deletion of predicted CREB binding sites #2 and #6 (Mut-2 and Mut-5) within the miR-373 promoter region reduced ZIP4-induced luciferase activity in pancreatic cancer cells". Aren't Mut-1 and Mut-3 statistically relevant in pancreatic cancer cells as well?
Response: The decision to focus on Mut-2 and Mut-5 was based on two experimental results: 1)

these mutants show the most profound effect in all the analyzed cell lines (MIA PaCa-2, AsPC-1, PL45 and HEK 293 cells) and 2) the effect of Mut-1 and Mut-3 sites was variable throughout all cell lines, especially in the pancreatic cancer cells, in some cases it did not show statistical differences with the control group (Supporting Information Fig. S2E, S2F and S2G). Therefore, we have decided to focus our studies on Mut-2 and Mut-5 as the CREB-responsive elements of this promoter.
This is reasonable. Would you please state this in the text.

5. In Fig.3C the authors show that inhibition of miR-373 expression leads to a decrease in cell proliferation. However, they do not provide an explanation for this phenomenon. Are they observing cell death or cell cycle arrest? Cell cycle distribution should be evaluated by flow cytometry. BrdU assay (to determine the percentage of cells in S phase) and Annexin V (to investigate apoptosis) should be performed as well. TUNEL assay is discouraged as miR-373 is involved in DNA damage response.
Response: As suggested by the reviewer, we have performed cell cycle analysis and determined the levels of apoptosis in MIA-ZIP4-Anti373 and MIA-ZIP4-AntiC cells by flow cytometry using BrdU and Annexin V staining. Our data indicate that blockade of miR-373 caused cell cycle arrest at G0/G1 phase without significant change in the apoptotic rate. The new data has been added in Supporting Information Fig. S3 as new Fig. S3A, S3B and S3C.
Would you please use the same scale for the Y-axis (Fig.S3A): this would make it easier for the reader to understand the data. As for Fig.S3B, this is not the most correct way to show BrdU incorporation because both cells in S-phase and in G2/M-phase would be BrdU positive. However, as the authors have shown that there are almost no cells in G2/M in their experimental setup, this way of presenting their data can be accepted. The authors should however mention this in the text.

6. In Fig.4, the authors injected TP53INP1, LATS2 and CD44 silenced MIA-PaCa-2 cells both subcutaneously and orthotopically and analyzed the tumor growth in this two systems and found that silencing of these proteins significantly increased the tumor growth and metastatization. By comparing the histological analysis of silenced tumors with the control ones, authors assessed that the percentage of the tumor area is higher in the former. Could the authors explain what they mean by "tumor area"? (Do they refer to the percentage of growing MIA-PaCa-2 cells respect to the stromal component?)
Response: Tumor area is defined as the percentage of tumor cells versus surrounding benign cells such as stromal, duct, and acinar cells (Histol Histopathol 25, 423-432 (2010) and World J Gastroenterol 2005;11(43):6765-6769). The tissue sections were stained with H&E, and the microscopic images were analyzed with NIH ImageJ to define the area of the tumor cells (tumor area) and the surrounding cells (none tumor area) (Fig. S4).
The point has been fully answered, maybe it is worth to mention it in the Materials and Methods section, or at least add "Histol Histopathol 25, 423-432 (2010) and World J Gastroenterol 2005;11(43):6765-6769" to the References.

9. In order to corroborate the data shown in Fig.7, percentage of positive Ki67 nuclei should be shown.
Response: We have added the quantitative data of Ki67 staining in Fig. 7, which indicates that silencing of the miR-373 target genes led to increased cell proliferation in the xenograft tumors. The Ki67 positive cells are 52% (shTP53INP1), 47% (shLATS2), and 65% (shCD44), respectively, compared with the 14% positive cells in the control group (shV).
The point has been properly answered. It would be suggested to perform Hematoxylin counterstaining to better discriminate Ki-67 positive from Ki-67 negative nuclei.

Referee #2 (Remarks):

The authors have adequately addressed almost all of my comments regarding the first submission. Unfortunately, their efforts to deal with one comment (Reviewer 2, point 1) has substantially undermined their hypothesis. They propose that Zip4 overexpression triggers increased miR-373 expression by increasing cytosolic zinc. However, new data presented in Figure S1F clearly contradicts that model. Specifically, increased miR-373 expression occurs when intracellular zinc is decreased by treatment with TPEN. These data indicate that Zip4 may be acting in a different way to

increase miR-373 expression.

Referee #3 (Remarks):

The statistical analysis in Figure 4 should be shown as individual mice data (plotted as individual points) with Median IQR (box and whisker) so that the reader can fully interpret the variability in this model system. This is hidden in Supplementary Figure. This version of data presentation has to be insisted.
The role of putative downstream role of TP53INP1, LATS2, and CD44 in growth and metastasis is unconvincing to be as emphatic in the lay summary and abstract. The conflicting hypothesis proposed in their previous paper (PNAS, Curr Mol Med and Clin Cancer Res) is not fully explained. The reader is left to belive that this is the only mechanism (Zip4-Creb-mir373-TP53INP1, LATS2, and CD44) whilst in fact the very same group has proposed (zip4-CREB4-Il6/STAT3 and zip4-VEGF/MMP) pathways which if this report is left in isolation is misleading. Suitable modification in abstract and discussion need to be made to bring these differing hypothesis together.

2nd Revision - authors' response 04 June 2013

Referee #1

1. The authors' response to the critique explaining the focus of the study on a subset of CREB sites is reasonable. Would you please state this response in the text?

Response: As suggested by the reviewer, we have included this response in the revised manuscript.

2. Would you please use the same scale for the Y-axis (Fig.S3A): this would make it easier for the reader to understand the data.

Response: We have re-adjusted the Y-axis to the same scale in Fig. S3A as recommended.

3. As for Fig.S3B, this is not the most correct way to show BrdU incorporation because both cells in S-phase and in G2/M-phase would be BrdU positive. However, as the authors have shown that there are almost no cells in G2/M in their experimental setup, this way of presenting their data can be accepted. The authors should however mention this in the text.

Response: We appreciate the suggestions by the reviewer, and have included a detailed explanation of the results of Fig. S3B in the supplementary information mentioning the low number of cells observed in G2/M phase in the cell cycle analysis, thus suggesting that the majority of the BrdU positive cells are in S phase.

4. Could the authors explain what they mean by "tumor area"? The point has been fully answered, maybe it is worth to mention it in the Materials and Methods section, or at least add "Histol Histopathol 25, 423-432 (2010) and World J Gastroenterol 2005;11(43):6765-6769" to the References.

Response: We have added the definition of tumor area in the Materials and Methods section, and included these two references in the revised manuscript.

5. The comment on the percentage of positive Ki67 nuclei has been properly answered. It would be suggested to perform Hematoxylin counterstaining to better discriminate Ki-67 positive from Ki-67 negative nuclei.

Response: We have performed Hematoxylin counterstaining after Ki67 staining in the same set of tissues, and included the new data in Fig. 7. The results indicate that silencing of the miR-373 target

genes led to increased cell proliferation in the xenograft tumors. The Ki67 positive cells are 52% (shTP53INP1), 47% (shLATS2), and 65% (shCD44), respectively, compared with the 14% positive cells in the control group (shV).

<u>Referee #2</u>

The authors have adequately addressed almost all of my comments regarding the first submission. Unfortunately, their efforts to deal with one comment (Reviewer 2, point 1) has substantially undermined their hypothesis. They propose that Zip4 overexpression triggers increased miR-373 expression by increasing cytosolic zinc. However, new data presented in Figure S1F clearly contradicts that model. Specifically, increased miR-373 expression occurs when intracellular zinc is decreased by treatment with TPEN. These data indicate that Zip4 may be acting in a different way to increase miR-373 expression.

<u>Response:</u> We apologize for the confusion. Below is our interpretation of these data that we believe is not in conflict with our conclusions and support the role of zinc in ZIP4-induced miR-373 expression.

In the zinc chelation experiment we treated ZIP4 overexpressing MIA-ZIP4 and control MIA-V cells using TPEN to deplete the intracellular zinc, then added dose-dependent $ZnCl_2$ back into the culture medium and examined the miR-373 expression in those cells before and after TPEN treatment. We demonstrate that the removal of zinc by TPEN treatment significantly decreased the miR-373 level in cells overexpressing ZIP4 (P<0.05), and has minimal impact on miR-373 in control MIA-V cells which have low ZIP4 expression (Fig. S1F). These data strongly indicate that zinc is necessary for the ZIP-mediated miR-373 expression. As pointed by the reviewer, we have also noted that the miR-373 level is still higher in MIA-ZIP4 cells than the ones in MIA-V control cells even after TPEN treatment without adding exogenous $ZnCl_2$. This might have two potential explanations: **1)** the TPEN chelation was incomplete and there were remaining zinc in the cells, **2)** we are detecting mature miR-373 not primary miR-373, and there is a delayed time for the mature miR-373 level to drop down after TPEN treatment because of the longer half-life of mature miRNA. Similar effects have been seen with other miRNAs under various treatments/conditions (Gantier MP et al. Analysis of microRNA turnover in mammalian cells following Dicer1 ablation. *Nucleic Acids Res*. 2011; Kai ZS et al., MicroRNA assassins: factors that regulate the disappearance of miRNAs. *Nat Struct Mol Biol*. 2010).

The results of these overexpression studies were further supported by the RNAi experiments showing that ZIP4 silencing causes decreased zinc induction of miR-373 (P<0.05, Fig. S1G) in AsPC-1 cells cultured with 0.5μM $ZnCl_2$. Please see below the supporting information Fig. S1F and S1G.

F

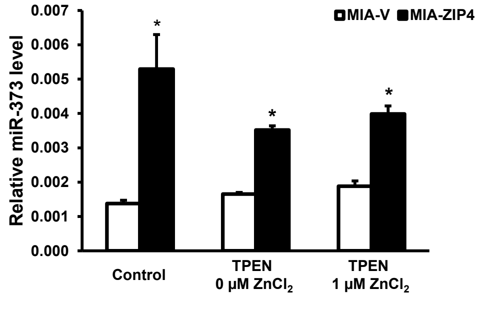

G

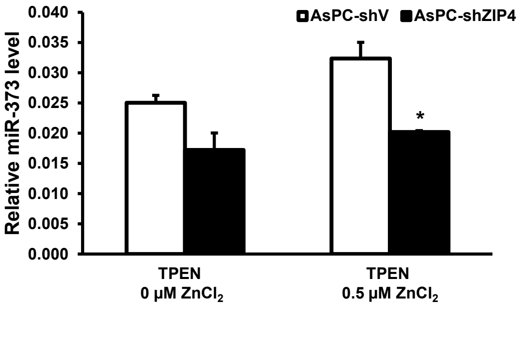

© EMBO

Similar to the miR-373 expression studies, our previous work (Li et al, PNAS 2007) also supports a zinc-dependent growth promoting effect of ZIP4 in MIA-ZIP4 cells. No significant increase of cell proliferation was found in MIA-V control cells upon addition of $ZnCl_2$. However, when a higher concentration of $ZnCl_2$ was added (~50 μM) to MIA-ZIP4 cells, cell proliferation was dramatically decreased, probably because of the toxicity of zinc. In contrast, MIA-V cells were less sensitive to zinc than MIA-ZIP4 cells, due to that MIA-V cells take up less zinc than MIA-ZIP4 cells. Those data indicate a zinc-dependent growth advantage of pancreatic cancer cells with ZIP4 overexpression.

Thus, together the results on zinc-dependent miR-373 expression in this study and our previous study on zinc-dependent cell proliferation do not completely rule out a zinc-independent effect of ZIP4, however, clearly support a role for zinc as an intermediate signaling molecule required in part by ZIP4 to modulate miR-373 in pancreatic cancer.

Again, we apologize for the misleading description of our data. To avoid further confusion, we have added the miR-373 expression data before TPEN treatment in MIA PaCa-2 cells to show that the removal of zinc by TPEN treatment significantly decreased the ZIP4 effect on miR-373 level to clearly show the involvement of zinc in the ZIP4-mediated expression of miR-373. We have also included an explanation of the data in the text (pages 4 and 5).

Referee #3

1. The statistical analysis in Figure 4 should be shown as individual mice data (plotted as individual points) with Median IQR (box and whisker) so that the reader can fully interpret the variability in this model system. This is hidden in Supplementary Figure. This version of data presentation has to be insisted.

Response: We have revised the box and whisker plot by including the individual mice data with median IQR (Fig. S4D). To avoid completely overlapped data, the individual data points are randomly jittered horizontally. The conclusion of the statistical analysis remains the same. Specifically, the statistical analysis result showed that: for the orthotopic tumor data, P=0.01 using two-sided t-test, and P=0.008 using two-sided Wilcoxon rank sum test; for the subcutaneous tumor data, P=0.004 using two-sided t-test, and P=0.008 using two-sided Wilcoxon rank sum test. As suggested by the reviewer, we have referred to this supplementary figure in the text which is highlighted in red.

2. The role of putative downstream role of TP53INP1, LATS2, and CD44 in growth and metastasis is unconvincing to be as emphatic in the lay summary and abstract. The conflicting hypothesis proposed in their previous paper (PNAS, Curr Mol Med and Clin Cancer Res) is not fully explained. The reader is left to belive that this is the only mechanism (Zip4-Creb-mir373-TP53INP1, LATS2, and CD44) whilst in fact the very same group has proposed (zip4-CREB4-Il6/STAT3 and zip4-VEGF/MMP) pathways which if this report is left in isolation is misleading. Suitable modification in abstract and discussion need to be made to bring these differing hypothesis together.

Response: We appreciate the reviewer's comment, and have revised the text to compare the current results with our previous publications (please see below).

Our group has recently demonstrated a novel biological role for the zinc importer ZIP4 in pancreatic cancer (Li 2009; Li 2007; Yang 2013; Zhang 2010), we have shown that ZIP4 is a major zinc importer upregulated in pancreatic cancer, and promotes cancer growth. Overexpression of ZIP4 may cause activated IL-6/STAT3 pathway and also led to increased VEGF and MMP expression, however, the detailed mechanism of how ZIP4 overexpression causes the activation of the downstream signaling pathways to promote pancreatic cancer growth is not clear. In this study, we

report a novel mechanism where increased zinc levels mediated by a zinc importer ZIP4 transcriptionally induces miR-373 in a CREB-dependent manner in pancreatic cancer cells. These results define a novel ZIP4-CREB-miR-373 signaling axis promoting pancreatic cancer growth, providing more in-depth mechanistic insights on how a zinc transporter functions in cancer cells and may have broader implications as inappropriate regulation of intracellular zinc concentration plays an important role in many other diseases.

We also realize that this may not be the only signaling pathway that mediates ZIP4-induced pancreatic cancer growth, other signaling pathways such as IL-6/STAT3 and pro-angiogenetic pathways may act in concert with miR-373 to regulate the common transcriptional effector (e.g. CREB).

The above paragraph has been included in the discussion section, which is highlighted in red. The abstract, discussion and summary of the results have been revised accordingly based on the reviewer's suggestions. We will be happy to expand on this discussion if the reviewer considers it is necessary.

3rd Editorial Decision 06 June 2013

Thank you for the submission of your revised manuscript to EMBO Molecular Medicine. We have now received the enclosed reports from the Reviewers that were asked to re-assess it. As you will see the reviewers are now supportive and I am pleased to inform you that we will be able to accept your manuscript pending the following final amendments:

1) As per our Author Guidelines, the description of all reported data that includes statistical testing must state the name of the statistical test used to generate error bars and P values, the number (n) of independent experiments underlying each data point (not replicate measures of one sample), and the actual P value for each test (not merely 'significant' or 'P < 0.05').

2) As you will see, Reviewer 3 has remaining requests. At this time, I will only ask you to comply with his/her request to make Fig. S4D a main figure. As this will imply some reorganisation of numbers and legends, please take the utmost care in doing this to avoid further revisons and delays. In any case, your manuscript will not be going back to the Reviewer.

3) The lettering on the heatmap in panel A of Fig.1 is not readable (and the heatmap itself is low resolution). Could you please provide a better image?

4) Please convert the red lettering (no longer needed) into black before you upload your new manuscript

Please submit your revised manuscript within two weeks. Needless to say, the sooner we receive it the sooner I will be able to formally accept your manuscript. Again, please make sure you double-check everything so that we can the proceed swiftly with formal acceptance.

I look forward to reading the revised final version of your manuscript as soon as possible.

***** Reviewer's comments *****

Referee #1 (General Remarks):

The authors have clarified all the issues raised by this reviewer.

Referee #2 (General Remarks):

none- the authors have addressed my last concern

Referee #3 (Comments on Novelty/Model System):

Whilst the model systems are adequate and the technical and reporting quality have improved, the novelty and the medical impact of the potential proposed mechanism has now been diluted.

Referee #3 (General Remarks):

Please move Figure S4D to main figure. Please report all in vivo data as Median +IQR in main figure with individual mice plotted.

2nd Revision - authors' response 10 June 2013

According to the reviewers' suggestions, we have moved the supplementary figure Fig. S4D to main figures as a new Fig. 4B, and provided the real P values for all statistical analysis. We also replace the Fig. 1A with a higher resolution figure.

We believe that the revised manuscript is significantly improved, and we hope that we have adequately addressed the important issues raised by the reviewers.
