# Supplementary material for: A novel epigenetic CREB-miR-373 axis mediates ZIP4-induced pancreatic cancer growth
Source: EMBO Mol Med. 2013 Jul 16;5(9):1322–34. doi: 10.1002/emmm.201302507 (PMC3799489; doi:10.1002/emmm.201302507)
Supplement: Supplementary file 2 [file emmm0005-1322-SD2.pdf]

Supporting Information

Zhang et al, A novel epigenetic CREB-miR373 axis mediates ZIP4-induced pancreatic cancer growth

Table of Contents:

Supporting Information Figure S1: ZIP4 induces the expression of miR-373 in pancreatic cancer.

Supporting Information Figure S2: The expression of miR-373 is regulated by the transcription factor CREB.

Supporting Information Figure S3: miR-373 promotes ZIP4-mediated pancreatic cancer cell invasion and proliferation.

Supporting Information Figure S4: miR-373 is required by ZIP4 to promote pancreatic cancer growth and metastasis.

Supporting Information Figure S5: TP53INP1, LATS2 and CD44 are target genes of miR-373 controlled by ZIP4.

Supporting Information Figure S6: miR-373 target genes mediate ZIP4-induced pancreatic cancer growth.

Supporting Information Figure S7: Summary of the metastasis.

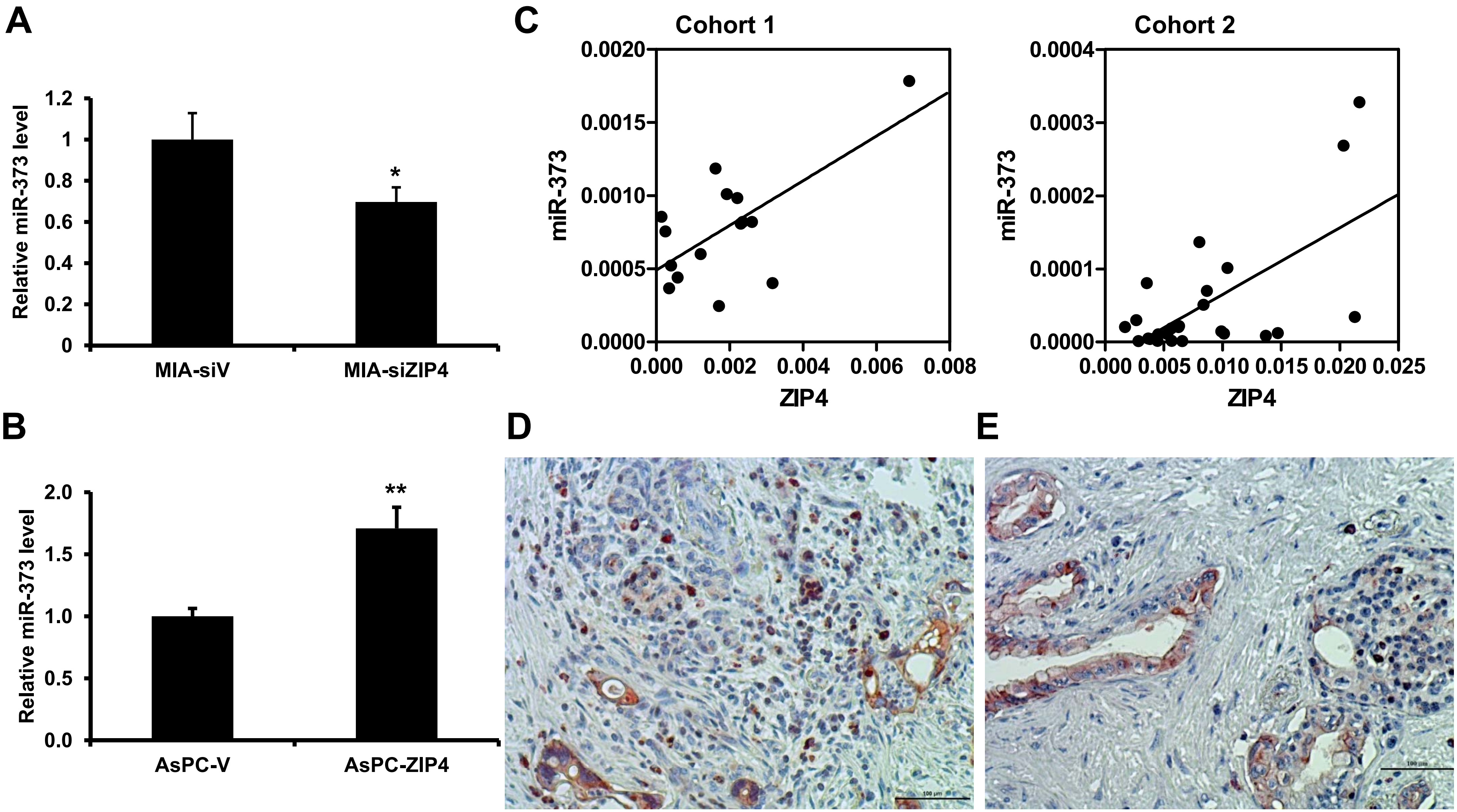

Fig. S1. ZIP4 induces the expression of miR-373 in pancreatic cancer. (A) Silencing of ZIP4 causes reduced expression of miR-373 in MIA PaCa-2 cell. (*P*=0.023, t-test, n=3). **(B)** Overexpression of ZIP4 upregulates miR-373 in AsPC-1 cell. Data were expressed as mean ± SD of triplicate values (*P*=0.0025, t-test, n=3). **(C)** Scatterplot of miR-373 expression vs ZIP4 expression in pancreatic cancer tissues from two independent cohorts (cohort 1, US cohort, n=15; cohort 2, Shanghai cohort, n=25). Pearson Correlation = 0.670, *P*=0.006 for cohort 1, and Pearson Correlation = 0.641, *P*=0.001 for cohort 2, respectively. Residual plots showed no evidence of lack of fit or substantial departures from normality. (**D), (E)** IHC staining of ZIP4 in two representative pancreatic cancer tissues (20X), showing the positive staining of ZIP4 in tumor cells, and negative staining in stroma, and normal duct cells. The scale bar is 100 µm.

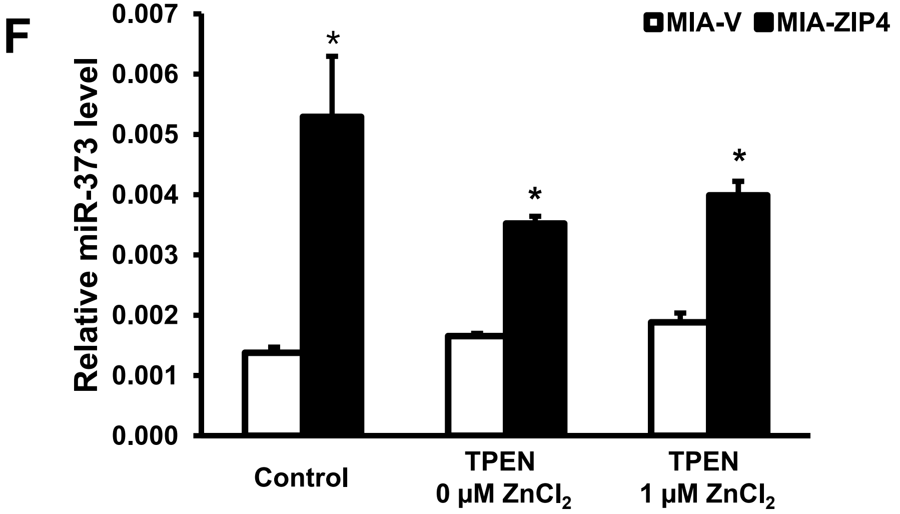

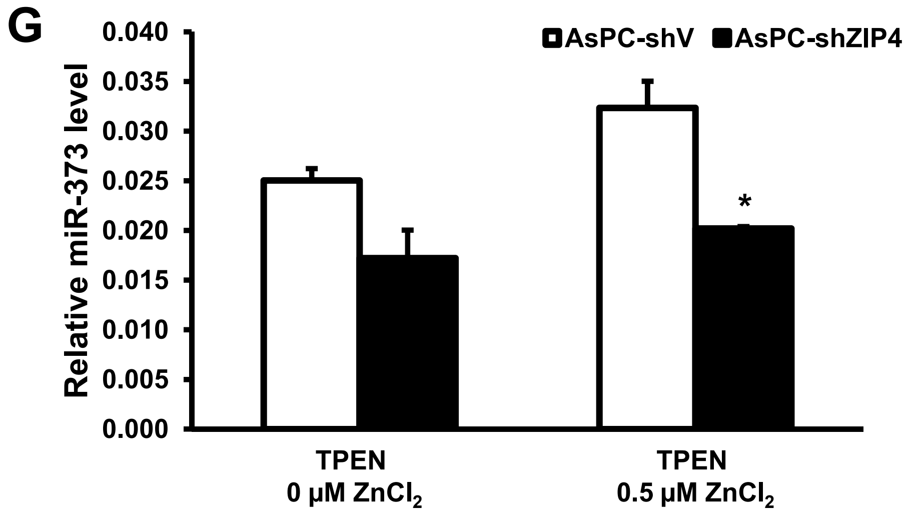

Fig. S1 (cont'd). ZIP4 induces the expression of miR-373 in pancreatic cancer. (F) Zinc-dependent upregulation of miR-373 in MIA-ZIP4 cells compared with MIA-V cells. Cells were treated with 4 µM TPEN (Life Technologies) for 1 hr, followed by DMEM incubation with different zinc concentration for 24 hrs. The expression of miR-373 were determined by real time PCR before (control) and after TPEN treatment (from left to right, P=0.0327, 0.0366, 0.043; t-test, n=3). **(G)** Zinc-dependent downregulation of miR-373 in AsPC-shZIP4 cells compared with AsPC-shV cells. The expression of miR-373 were determined by real time PCR as above. Data were expressed as mean $\pm$ SD of triplicate values (significant P value =0.0462, t-test, n=3).

A

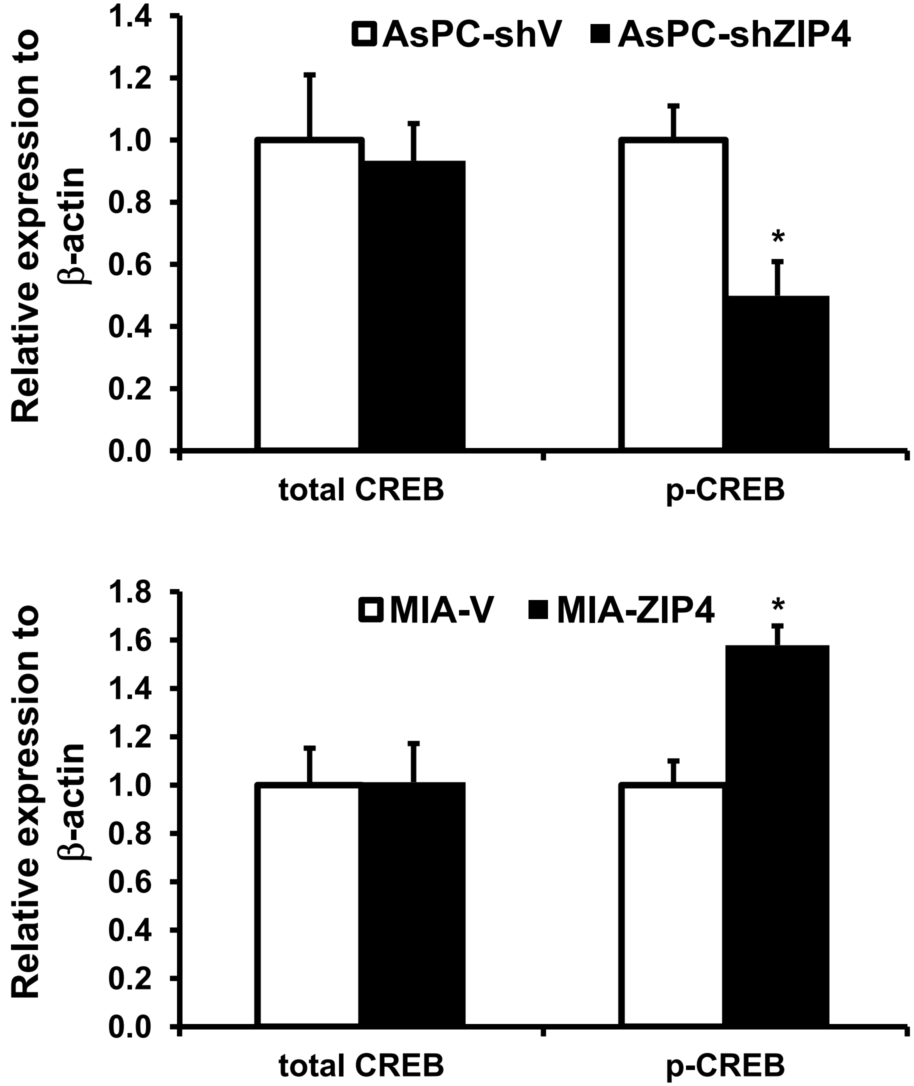

Fig. S2. The expression of miR-373 is regulated by the transcription factor CREB. (A) Densitometry analysis of the WB results in Fig. 2A (from top to bottom, significant *P* value =0.0412, 0.0203; t-test, n=3). Data were expressed as the mean ± SD of triplicate values.

B

miR-373 Promoter

	Transcription factor	Sequence	From	To	Score	Strand
7	CREB	CATGTTGGCGTT	683	694	7.029	+
6	CREB	GGTGTG**~~GACG~~**TA	927	938	7.935	+
5	CREB	CAGGAT**~~GAAG~~**TG	1023	1034	7.836	+
4	CREB	GATGATGGCGCC	1044	1055	7.546	+
3	CREB	AG**~~CGTCA~~**CCGGT	1883	1894	7.827	-
2	CREB	ACCGG**~~TGAC~~**GCC	1889	1900	9.049	+
1	CREB	GGC**~~GTGA~~**ACCCG	2329	2340	7.593	-

C

**Primer**	**Mutation Type**	**Deletion loci**	**Sequence 5' to 3'**	**Targeted CREB sites**
Mut-1	GTGA	del5106-5109	CAGGAGAATGGC***ACCCGGGAGGCG***	1# CREB site
		del5106-5109-antisense	**CGCCTCCCGGGT**GCCATTCTCCTG	GGC**~~GTGA~~**ACCCG
Mut-2	TGAC	del4668-4671	TTGAGCGTCACCGG**GCCCATATCAACGG**	2# CREB site
		del4668-4671-antisense	**CCGTTGATATGGGC**CCGGTGACGCTCAA	ACCGG**~~TGAC~~**GCC
Mut-3	CGTCA	del4659-4663	CTGCGACATTTGAG**CCGGTGACGCCCA**	3# CREB site
		del4659-4663-antisense	**TGGGCGTCACCGG**CTCAAATGTCGCAG	AG**~~CGTCA~~**CCGGT
Mut-4	GAAG	del3803-3806	CTCACTGTCGCCCAGGAT**TGCACAGGTAG**	5# CREB site
		del3803-3806-antisense	**CTACCTGTGCA**ATCCTGGGCGACAGTGAG	CAGGAT**~~GAAG~~**TG
Mut-5	GACG	del3707-3710	GCAGACCTGAGGTGTG**TATCATTGGCCTCTGT**	6# CREB site
		del3707-3710-antisense	**ACAGAGGCCAATGATA**CACACCTCAGGTCTGC	GGTGTG**~~GACG~~**TA

Fig. S2 (cont'd). The expression of miR-373 is regulated by the transcription factor CREB. (B) Predicted CREB binding sites in miR-373 promoter region. **(C)** Mutation primers of CREB binding sites in miR-373 promoter region.

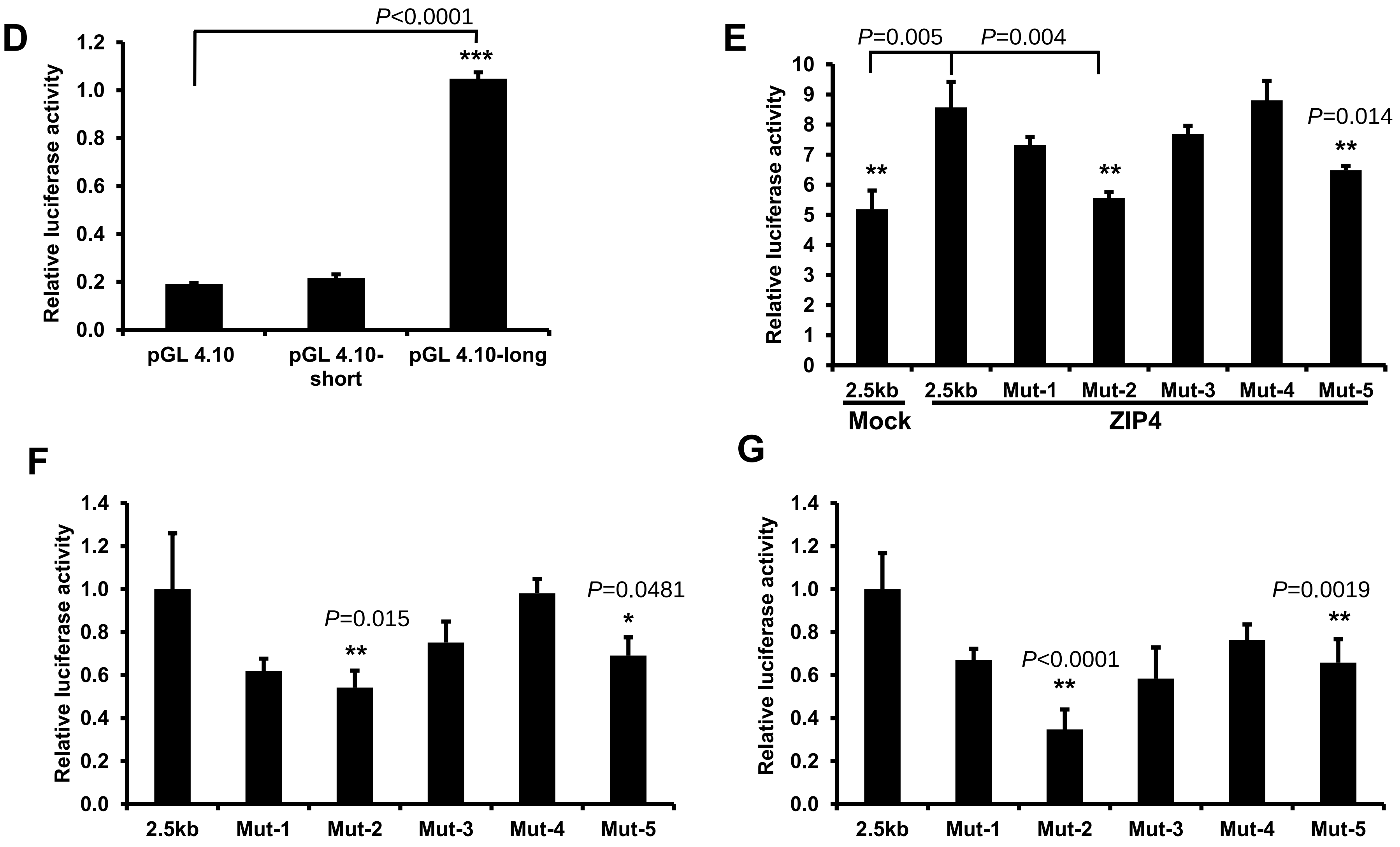

Fig. S2 (cont'd). The expression of miR-373 is regulated by the transcription factor CREB. (D) Promoter activity. The promoter reporter construct was co-transfected with control plasmid pRL-TK into HEK293 cells, the promoter activity was determined by a chemiluminescence reader (t-test, n=3). **(E), (F)** and **(G)** Mutational analysis of miR-373 promoter. The wild type and mutant promoter constructs were co-transfected with control plasmid pRL-TK into (E) HEK293 (plus mock or ZIP4 vector) (t-test, n=3), (F) MIA PaCa-2 (t-test, n=4) or (G) PL45 cells (t-test, n=6). Data were expressed as mean ± SD of triplicate values.

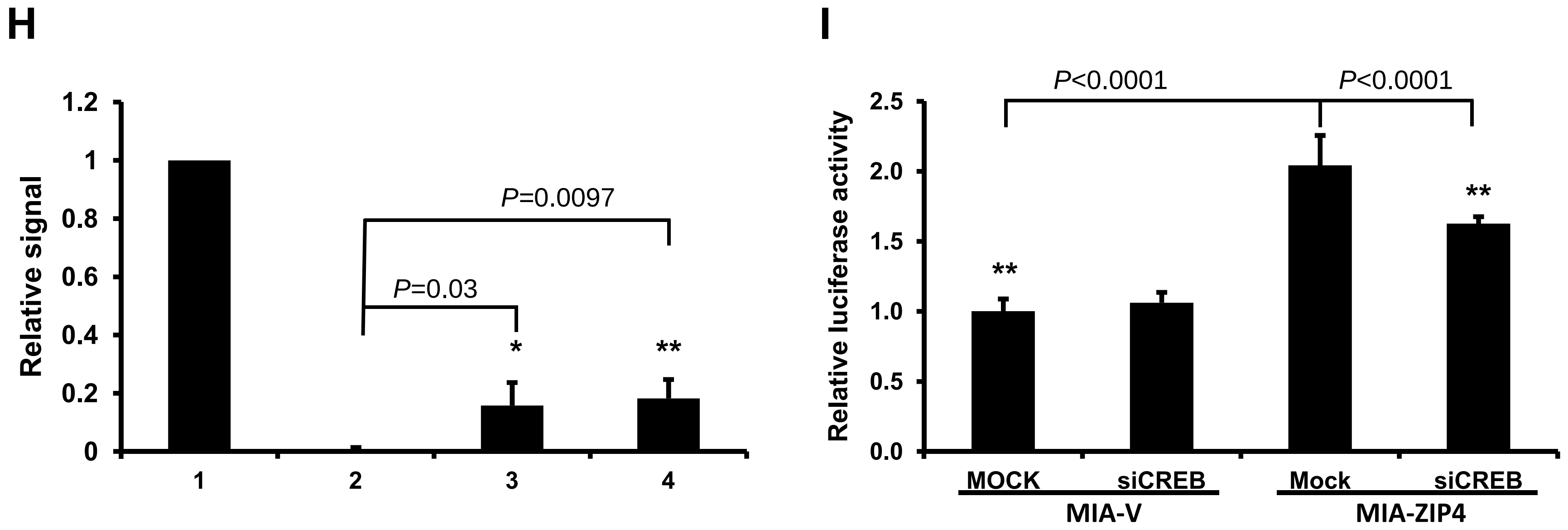

Fig. S2 (cont'd). The expression of miR-373 is regulated by the transcription factor CREB. (H) Densitometry analysis of the ChIP assay in Fig. 2E. 1, Input, 2, IgG control, 3, Anti-CREB, 4, Anti-p-CREB (t-test, n=3). **(I)** Luciferase activity was increased in MIA-ZIP4 cells compared with that in the control MIA-V cells, and was downregulated when CREB was silenced by siRNA (t-test, n=9). All data were shown as mean ± SD of triplicate values.

A

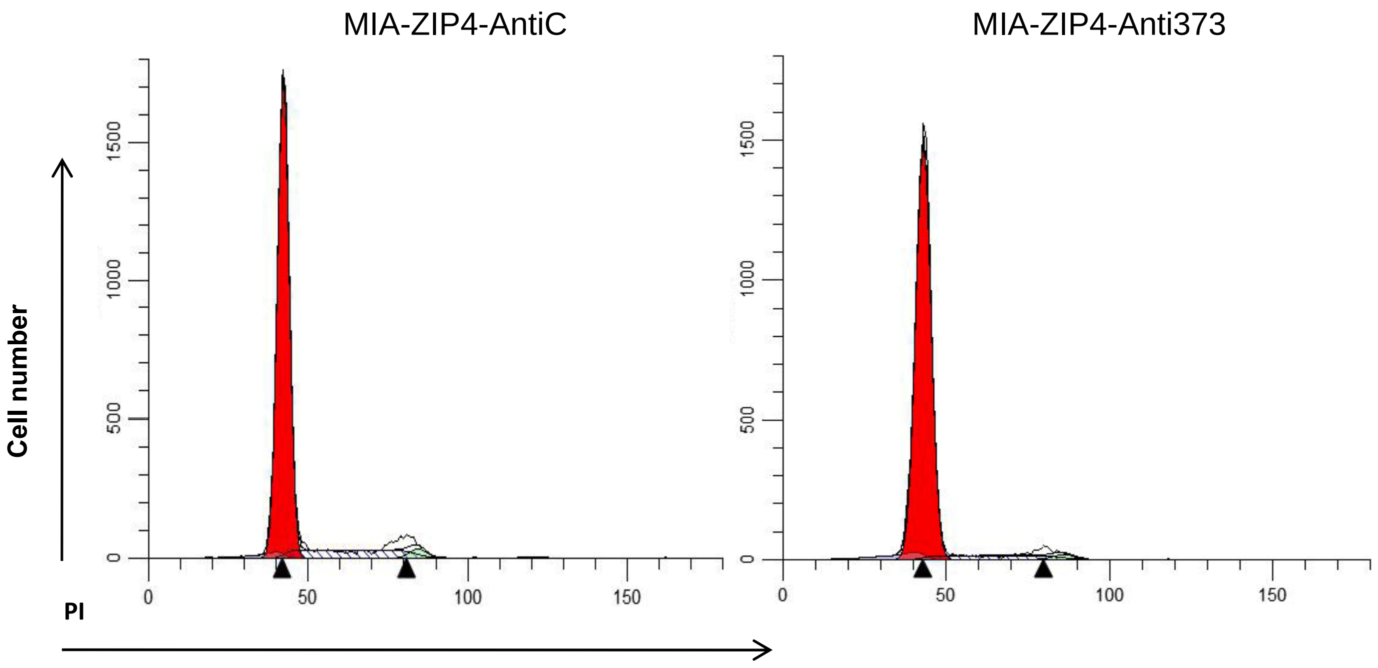

	% G1**	% S**	% G2/M
MIA-ZIP4-AntiC	85.5±1.3516	14.5±1.9516	0
MIA-ZIP4-Anti373	92.84±1.0182	7.0±0.9051	0.11±0.0424

Fig. S3. miR-373 promotes ZIP4-mediated pancreatic cancer cell invasion and proliferation. (A) Cell cycle analysis by flow cytometry. Cells were starved for 48 hrs and then released by incubation with FBS 10% DMEM medium for 2 hrs. Then cells were stained with PI (BD Bioscience) before acquisition by a flow cytometer (BD LSRII). The percentage of cells in G1, S and G2/M phases was measured and shown as mean ± SD of triplicate values (for G1 and S phase, *P*=0.0015, 0.0013, t-test, n=3).

B

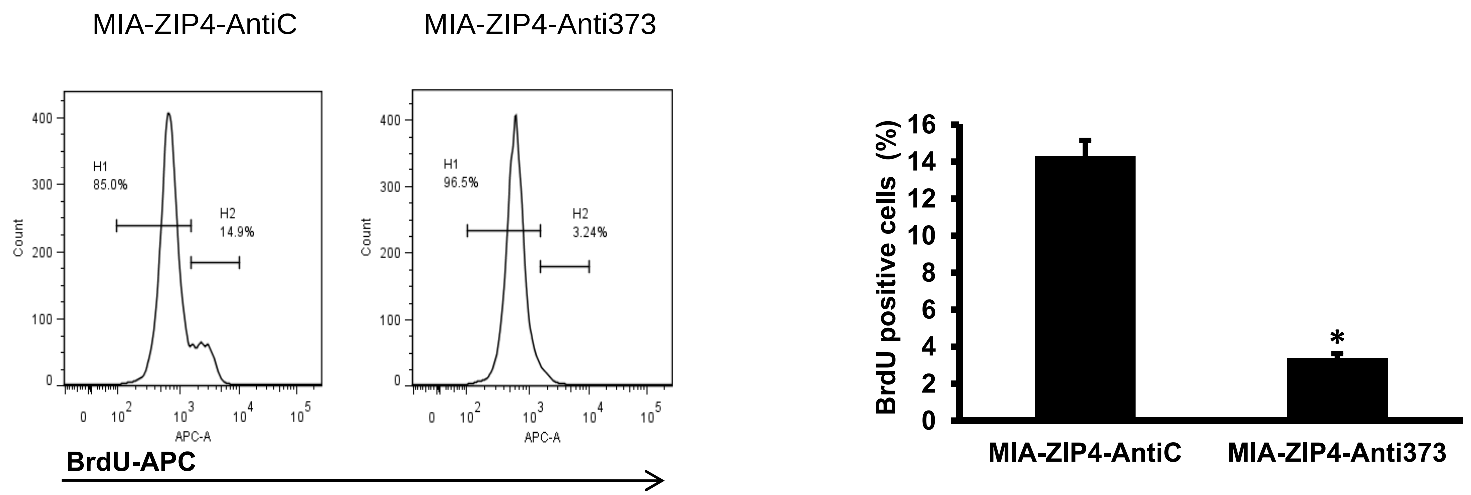

C

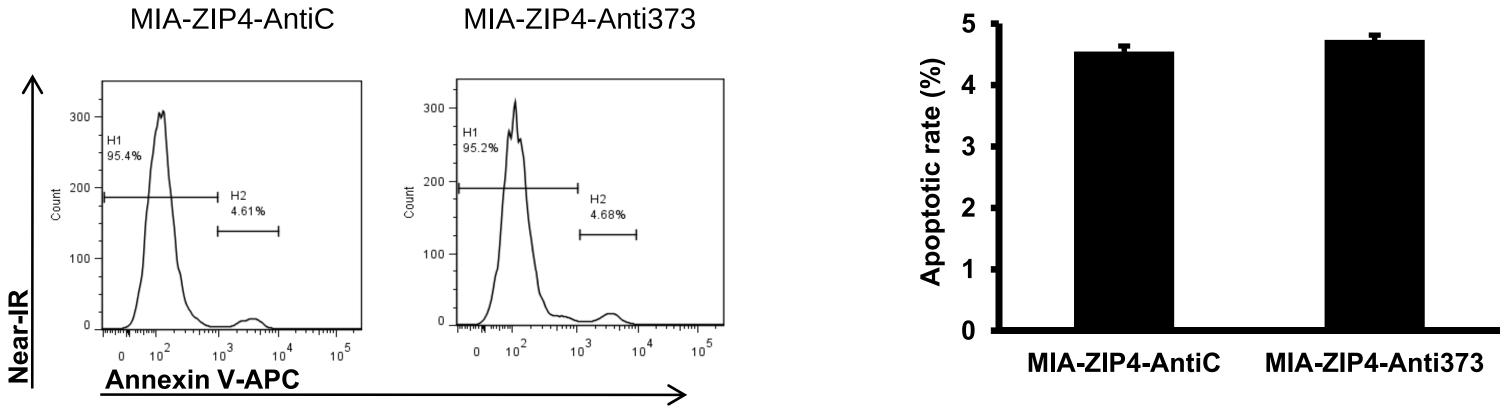

Fig. S3 (cont'd). miR-373 promotes ZIP4-mediated pancreatic cancer cell invasion and proliferation. (B) BrdU incorporation analysis by flow cytometry. Cells were starved for 48 hrs and then released by incubation with DMEM medium with 10% FBS for 2 hrs. Cells were treated with 10 µM APC labeled BrdU for 10 mins and 1:50 anti-BrdU Ab (BD Bioscience), then gated by cell width and area during acquisition by a flow cytometer. H1 stands for BrdU negative cells; since both cells in S-phase and G2/M-phase would be BrdU positive by single staining, and Fig. S3A has shown that almost no cells were observed in G2/M phase, H2 mainly represents BrdU positive cells in S phase. Representative histograms are shown indicating the BrdU positive cells ratio in MIA-ZIP4-AntiC (Left) and MIA-ZIP4-Anti373 (Right) cells. The quantitative data were also shown (P=0.032, t-test, n=3). **(C)** Apoptosis analysis by Annexin V assay. Cells were harvested with trypsin and incubated with APC labeled Annexin-V Ab (BD Bioscience). Cells were then doubly stained by Near-IR fluorescent reactive dye (Invitrogen), and excited separately with 633nm and 775nm laser in a BD flow cytometer. H1 stands for live cells, and H2 stands for apoptotic cells (P=0.1553, t-test, n=3). All data were shown as mean $\pm$ SD of triplicate values.

D

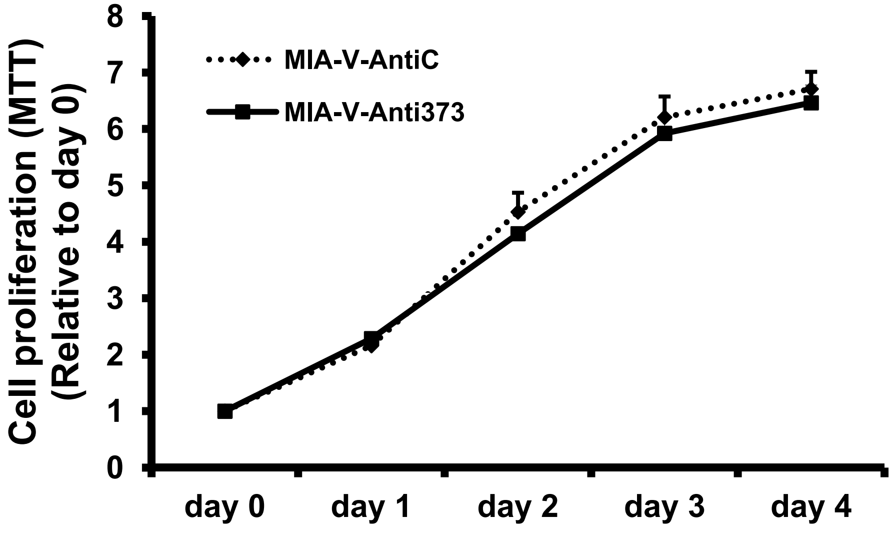

E

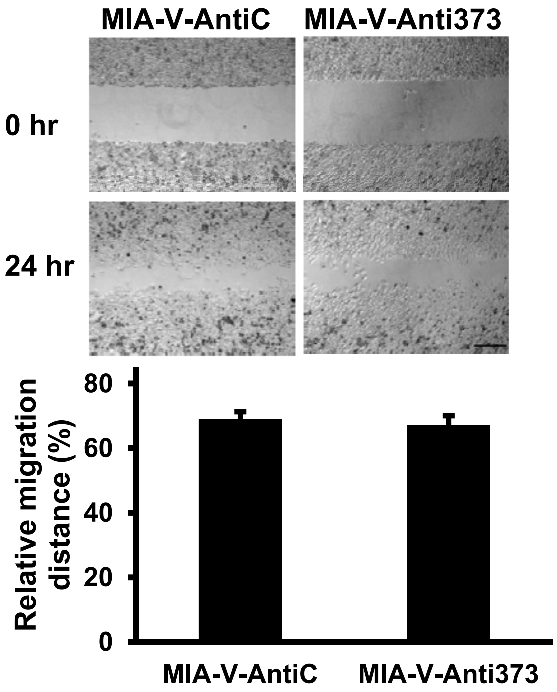

Fig. S3 (cont'd). miR-373 promotes ZIP4-mediated pancreatic cancer cell invasion and proliferation. (D) Cell proliferation was measured by MTT assay in MIA-V-AntiC and Anti373 cells. All data were standardized compared with day 0. **(E)** Wound healing assay in MIA-V-AntiC and Anti373 cells. Cells were treated with 10ug/ml mitomycin (Sigma) for 2 hrs. A representative field of wound healing was captured and recorded at 0 hr and 24 hrs. All data were shown as mean ± SD of triplicate values (*P*=0.5994, t-test, n=3). Scale bar equals to 500 µm.

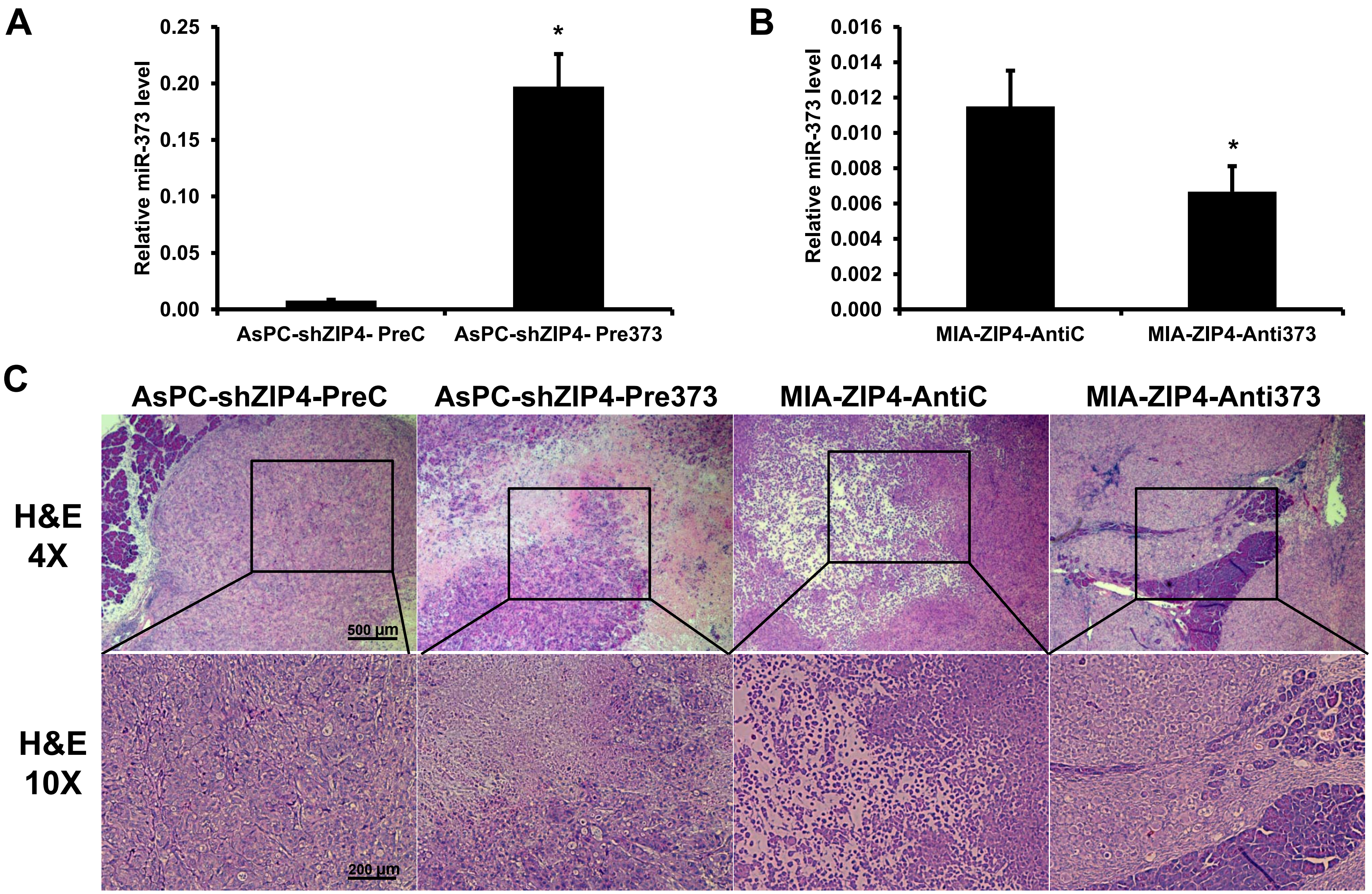

Fig. S4. miR-373 is required by ZIP4 to promote pancreatic cancer growth and metastasis. Real time RT-PCR showed that miR-373 was significantly increased in **(A)** AsPC-shZIP4-Pre373 cells (P<0.0001, t-test, n=3), and was reduced in **(B)** MIA-ZIP4-Anti373 cells (P=0.0342, t-test, n=3); data were expressed as mean ± SD of triplicate values. **(C)** H&E staining with xenograft tumor sections in 4X and 10X magnification, the scale bars are 500 µm (4X) and 200 µm (10X).

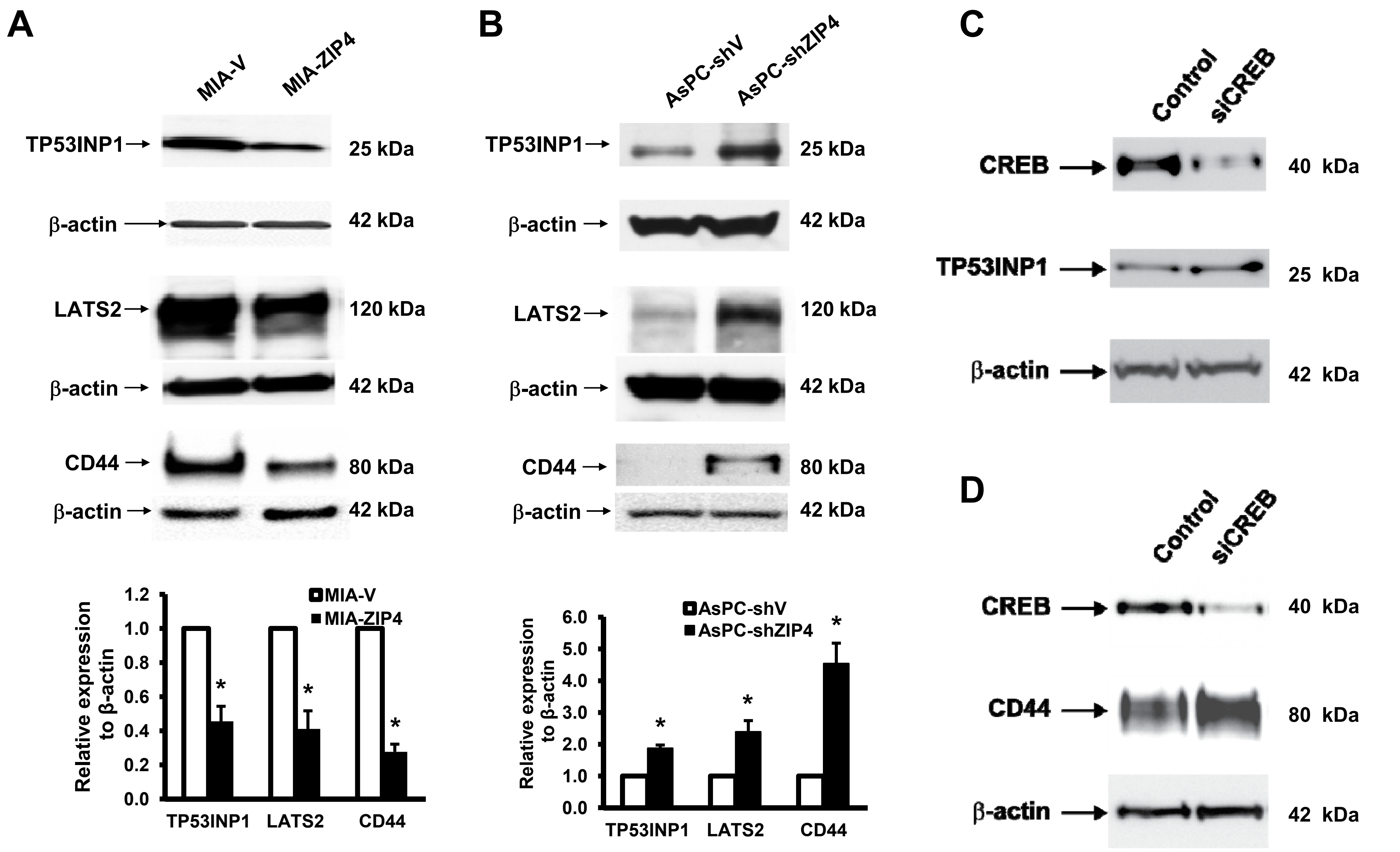

Fig. S5. TP53INP1, LATS2 and CD44 are target genes of miR-373 controlled by ZIP4. Expression of TP53INP1, LATS2 and CD44 in **(A)** MIA PaCa-2 (from left to right, *P*=0.0086, 0.0108, 0.0013, t-test, n=3), and **(B)** AsPC-1 cells (from left to right, *P*=0.0043, 0.01987, 0.0107; t-test, n=3). All data were shown as mean ± SD of triplicate values. Silencing of CREB leads to increased expression of miR-373 downstream target genes in **(C)** AsPC-1 and **(D)** MIA PaCa-2 cells. Cells were transfected with siRNA against CREB (Mission siRNA from Sigma) with the XtremeGENE 9 (Roche) following the standard protocol. The total cell lysates were assayed for western blot at 48 hrs post transfection.

E **miR-373** 3’ UGUGAGUUUGUACCUUCGUGAAU 5’

TP53INP1

Organism	miR-373 site
Homo sapiens	5’ CAAUACAGCACUUUGCCA 3’
Pan troglodytes	5’ CAAUACAGCACUUUGCCA 3’
Macaca mulatta	5’ CAAUACAGCACUUUGCCA 3’
Mus musculus	5’ CAGCACAGCACUUUGCCA 3’
Rattus norvegicus	5’ CAGCACAGCACUUUGCCA 3’
Cavia porcellus	5’ CAAUACAGCACUUUGCCA 3’
Oryctolagus cuniculus	5’ CAAUACAGCACUUUGCCA 3’
Sorex araneus	5’ CAGAACAGCACUUUGCCA 3’
Erinaceus europaeus	5’ UAAUACAGCACUCUGCCA 3’
Canis familiaris	5’ CAAUACAGCACUUUGCCA 3’
Equus caballus	5’ CAAUACAGCACUUUGCCA 3’
Bos taurus	5’ CAAUACAGCACUUUGCCA 3’

F **miR-373** 3’ UGUGAGUUUGUACCUUCGUGAA 5’

LATS2

Organism	miR-373 site-1	miR-373 site-2
Homo sapiens	5' GAAAGAGCACUUUU 3'	5' UCCAUAGCACUUUUCA 3'
Pan troglodytes	5' GAAAGAGCACUUUU 3'	5' UCCAUAGCACUUUUCA 3'
Macaca mulatta	5' GAAAGAGCACUUUU 3'	5' UCCAUAGCACUUUUCA 3'
Otolemur garnetti	5' GAAAGAGCACUUUU 3'	5' UCCAUAGCACUUUUCA 3'
Tupaia belangeri	5' GAAAGAGCACUUUU 3'	5' UCCAUAGCACUUUUCA 3'
Oryctolagus cuniculus	5' GAAAGAGCACUUUU 3'	5' UCCGUAGCACUUUUCA 3'
Canis familiaris	5' GAAAGAGCACUUUU 3'	5' UCCAUAGCACUUUUCA 3'

G **miR-373** 3’ UGUGAGUUUGUACCUCGUGAA 5’

CD44

Organism	miR-373 site
Homo sapiens	5' GUUGAAGCACUUUUGG 3'
Pan troglodytes	5' GUUGAAGCACUUUUGG 3'
Macaca mulatta	5' GUUGAAGCACUUUUGG 3'
Oryctolagus cuniculus	5' GUUGAAGCACUUUUGG 3'
Canis familiaris	5' GUUGAAGCACUUUAGG 3'
Felis catus	5' GUUGAAGCACUUUAGG 3'
Bos taurus	5' GCCGGAGCACUUUUGG 3'
Echinops telfairi	5' GAGGAAGCACUUUUGG 3'

Fig. S5 (cont’d). TP53INP1, LATS2 and CD44 are target genes of miR-373 controlled by ZIP4. Predicted miR-373 binding sites in the 3'UTR regions of potential target genes: TP53INP1 **(E)**, LATS2 **(F)**, and CD44 **(G)**.

H

FTP53INP1DE	CATAGATGGTCAGTTTTGTACACAGACTGAACAAT**ACTG**CCAAAAATGAGTGTAGCATTGTTTAAACATTGTGTG
RTP53INP1DE	CACACAATGTTTAAACAATGCTACACTCATTTTTGG**CAGT**ATTGTTCAGTCTGTGTACAAAACTGACCATCTATG
FCD44DE	GAGGAAAGCATGATATGTATATTGCTGAGTTG**AATT**GGAAAATATTAAAAGGCTAACATTAAAAGACTAAAGG
RCD44DE	CCTTTAGTCTTTTAATGTTAGCCTTTTAATATTTTCC**AATT**CAACTCAGCAATATACATATCATGCTTTCCTC
FLATS2DE-2	CATGCTGCTGTTATTTTCTACATTTGTATTTTATCC**ATTT**CACATTTAGGAAAAGACATAAAAACTGAAGAACATTG
RLATS2DE-2	CAATGTTCTTCAGTTTTTATGTCTTTTCCTAAATGTG**AAAT**GGATAAAATACAAATGTAGAAAATAACAGCAGCATG
FLATS2DE-1	CAATATTTTTGAAAATTTAGTACAGTTTAGAAAG**AGTA**TTTTGTTTATATCCATTTTTTCTTACTAAATTATAG
RLATS2DE-1	CTATAATTTAGTAAGAAAAAATGGATATAAACAAAA**TACT**CTTTCTAAACTGTACTAAATTTTCAAAAATATTG

Fig. S5 (cont'd). TP53INP1, LATS2 and CD44 are target genes of miR-373 controlled by ZIP4. (H) Mutation primer sequences of 3'UTR regions of miR-373 target genes. The putative miR-373 biding sites were abolished by site directed mutagenesis.

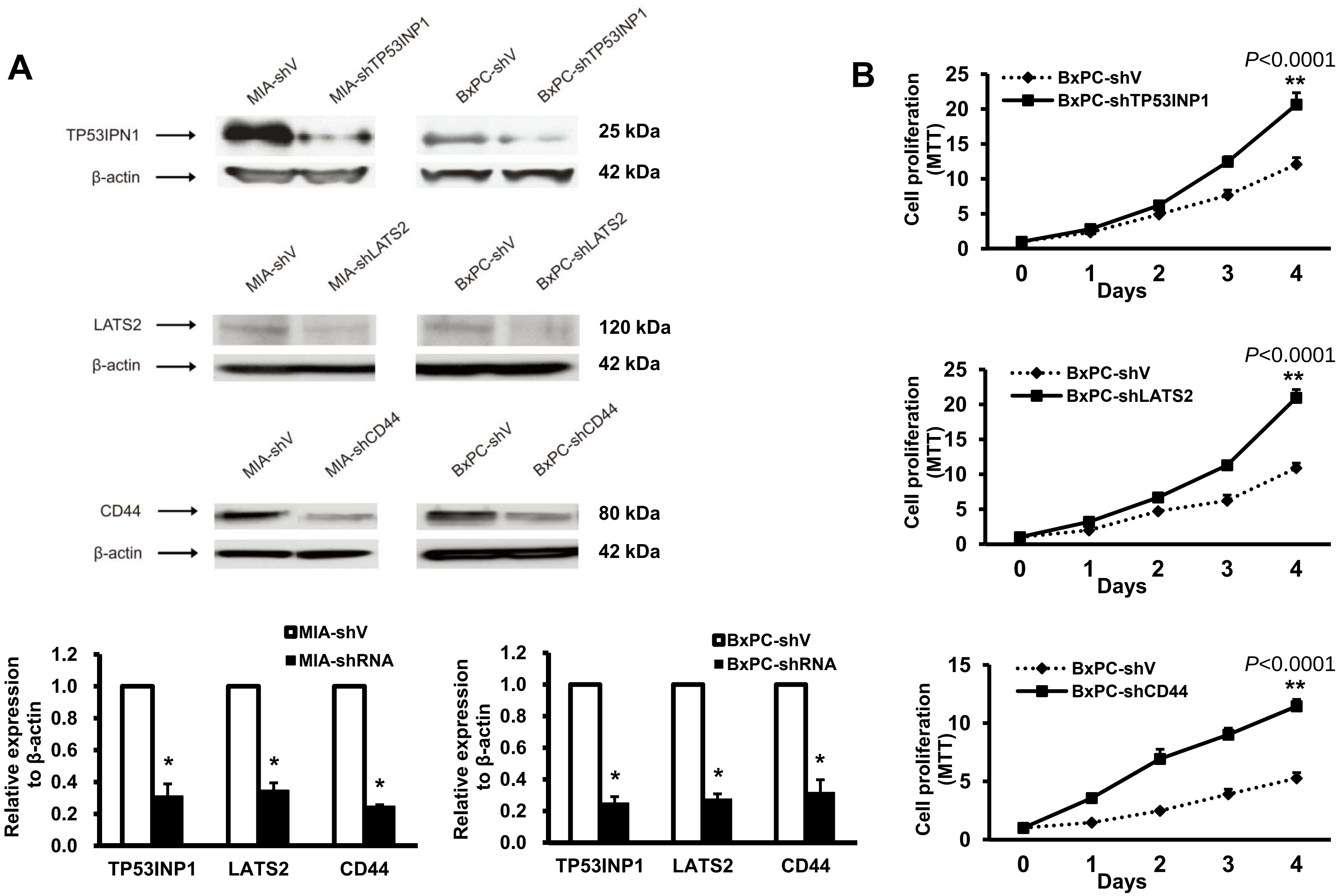

A
MIA-shV
MIA-shTP53INP1
BxPC-shV
BxPC-shTP53INP1
TP53IPN1
β-actin
25 kDa
42 kDa
MIA-shV
MIA-shLATS2
BxPC-shV
BxPC-shLATS2
LATS2
β-actin
120 kDa
42 kDa
MIA-shV
MIA-shCD44
BxPC-shV
BxPC-shCD44
CD44
β-actin
80 kDa
42 kDa
MIA-shV
MIA-shRNA
Relative expression to β-actin
TP53INP1
LATS2
CD44
BxPC-shV
BxPC-shRNA
Relative expression to β-actin
TP53INP1
LATS2
CD44
B
P<0.0001
**
BxPC-shV
BxPC-shTP53INP1
Cell proliferation (MTT)
Days
P<0.0001
**
BxPC-shV
BxPC-shLATS2
Cell proliferation (MTT)
Days
P<0.0001
**
BxPC-shV
BxPC-shCD44
Cell proliferation (MTT)
Days

C

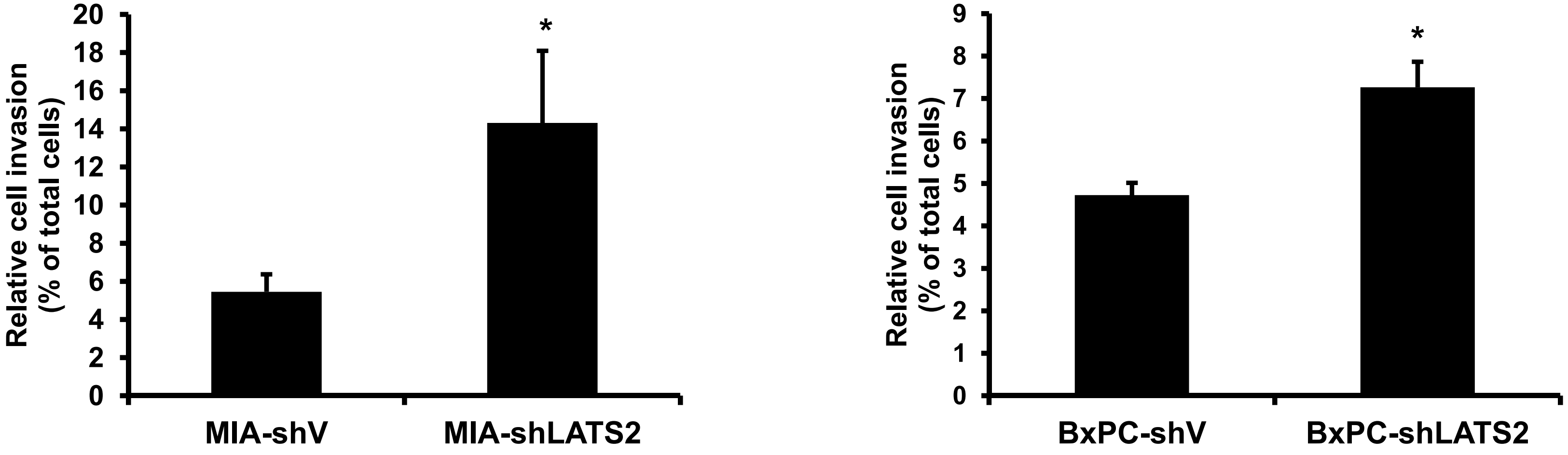

Fig. S6. miR-373 target genes mediate ZIP4-induced pancreatic cancer growth. (A) Western blot confirmed the knockdown of TP53INP1, LATS2, and CD44 in both MIA PaCa-2 and BxPC-3 cells (from left to right, *P*=0.0122, 0.0051, 0.0002, 0.0203, 0.0016, 0.0153*;* t-test, n=3). **(B)** Cell proliferation in BxPC-shTP53INP1, BxPC-shLATS2, and BxPC-shCD44 cells. Cell proliferation was determined by MTT assay and compared with day 0 value (t-test, from top to bottom, n=5, 4, 5). **(C)** Migration of MIA-shLATS2 and BxPC-shLATS2 cells were increased upon the knockdown of LATS2 (from left to right, *P*=0.011, 0.027; t-test, n=3). Data were expressed as the mean ± SD of triplicate values.

	MIA-shV	MIA-shLATS2	MIA-shTP53INP1	MIA-shCD44
No. of mice with ascites	1	2	3	2
No. of mice with peritoneal dissemination	5	7	7	9
No. of mice with liver metastasis	1	2	3	6
No. of mice with spleen metastasis	5	7	11	6
No. of mice with colon metastasis	1	5	8	5
Total no. of mice	10	9	12	10

Fig. S7. Summary of the metastasis. The major symptoms of the nude mice with xenografts of MIA-shTP53INP1, MIA-shLATS2, and MIA-shCD44 cells were summarized and compared to that of the control group MIA-shV.
